# Color variations during digital imaging of facial prostheses subjected to unfiltered ambient light and image calibration techniques within dental clinics: An in vitro analysis

**DOI:** 10.1371/journal.pone.0273029

**Published:** 2022-08-29

**Authors:** Farah Rashid, Nafij Bin Jamayet, Taseef Hasan Farook, Matheel AL-Rawas, Aparna Barman, Yanti Johari, Tahir Yusuf Noorani, Johari Yap Abdullah, Sumaiya Zabin Eusufzai, Mohammad Khursheed Alam

**Affiliations:** 1 School of Dental Sciences, Universiti Sains Malaysia, Kota Bharu, Kelantan, Malaysia; 2 Division of Restorative Dentistry, School of Dentistry, International Medical University, Kuala Lumpur, Malaysia; 3 Adelaide Dental School, The University of Adelaide, Adelaide, SA, Australia; 4 Prosthodontic Unit, School of Dental Sciences, Universiti Sains Malaysia, Health Campus, Kubang Kerian, Kota Bharu, Kelantan, Malaysia; 5 Conservative Dentistry Unit, School of Dental Sciences, Universiti Sains Malaysia, Health Campus, Kubang Kerian, Kota Bharu, Kelantan, Malaysia; 6 Craniofacial Imaging and Additive Manufacturing Laboratory, School of Dental Sciences, Universiti Sains Malaysia, Kota Bharu, Kelantan, Malaysia; 7 Orthodontics, Department of Preventive Dental Science, College of Dentistry, Jouf University, Sakaka, Saudi Arabia; Danube Private University, AUSTRIA

## Abstract

**Background:**

The study aimed to evaluate 1) the amount of color variations presents within clinical images of maxillofacial prosthetic silicone specimens when photographed under different clinically relevant ambient lighting conditions, and 2) whether white balance calibration (WBC) methods were able to mitigate variations in ambient lighting.

**Methods:**

432 measurements were acquired from standardized images of the pigmented prosthetic silicone specimens within different ambient lighting conditions (i.e., 2 windowed and 2 windowless clinics) at noon with no light modifying apparatus. The specimens were photographed once without any white balance calibration (raw), then independently alongside an 18% neutral gray card and Macbeth color chart for calibration in a post-processing (PPWBC) software, and once after camera calibration (CWBC) using a gray card. The LAB color values were extracted from the images and color variations (ΔE) were calculated after referring to the corresponding spectrophotometric values as control.

**Results:**

Images in windowless and windowed clinics exhibited highly significant differences (p < 0.001) with spectrophotometer (control). CWBC demonstrated no significant differences (p > 0.05) in LAB values across windowed clinics. PPWBC using Macbeth color chart produced no significant differences for a* values (p > 0.05) across all clinics while PPWBC by gray card showed no significant differences (p > 0.05) in LAB values when only similar clinics (either windowed or windowless) were compared.

**Conclusion:**

Significant color variations were present for maxillofacial prosthetic specimens owing to natural ambient light. CWBC and PPWBC using color charts were more suitable for color correction across windowed clinics while CWBC and PPWBC using gray cards had better outcomes across windowless setups.

## Introduction

Clinical photographs and color accurate imaging are crucial in digital dentistry, as photographs act as communication media between the practitioners and technicians [1–3]. Photographs also influence patient’s perceptibility (*i*.*e*., *ability to identify color differences*) and acceptability (*i*.*e*., *whether the color is acceptable by the observer*) [4] towards the treatment outcome by directly involving them into the treatment process and educating them on when a facial prosthesis replacement is clinically indicated [5].

Unfortunately, variations in ambient or environmental lights introduce several limitations for clinics without access to natural light (windowless clinics) and even more so for clinics with windows receiving natural light [6]. To mitigate these limitations, several investigators [7, 8] have suggested the use of electronic strobe lights which are generally found within professional photo studios as these lights are able to correct the white balance (*i*.*e*., *procedure to adjust color according to the light source available so that the color of the object appear natural within the photographs*) of the images. However, dental practitioners are generally limited by the inaccessibility to such professional tools and therefore take photographs without any added light modifiers [9].

Other options of professional color calibration through white balance corrections have been proposed in the past such as camera white balance calibration (CWBC) [10] and computerized software-based post-processing white balance calibration (PPWBC) [5]. These methods require tools ranging from inexpensive single color calibration cards (ex. Gray cards) to expensive multi-color calibration systems (ex. Macbeth color charts) [11]. When these systems are applied onto raw images produced by a camera with fixed manual settings, the light exposed within the scene will be balanced against the extra colors that might otherwise appear during the image capture (i.e., extra-global effect), thereby resulting in consistently accurate colors [12]. However, their effects on correcting color of facial prostheses in dental clinics remains unexplored.

In dentistry, visual color detection tools like shade guides are most widely used but the results are subjective, biased and adversely affected by the factors such as eye fatigue [1, 13]. In contrast, contact-measuring electronic color detection tools such as spectrophotometers are said to produce consistently accurate color results by measuring the light that is reflected from an object at about 1 to 25 mm interval together with a wavelength of light [14]. Previously, investigations [15, 16] had compared both procedures and determined that visual analyses tend to select darker shades and produce less reproducible color results than the spectrophotometer. However, spectrophotometers are expensive, have varying degrees of inter-device reliability, and are not readily available within most dental clinics [1, 3, 17–19]. Regardless, values from these devices are widely accepted as standard reference and they measure color values within the CIELAB color space [14, 17, 20, 21].

Amidst the various proposed color spaces for use in digital shade matching, in 1976, the CIE (International Commission on Illumination) proposed the CIELAB color space which was quickly adapted as standard [22]. This color space consists of 3 color co-ordinates: L, a* and b*. Here, L represents the lightness or darkness of the images, a* represents red to green co-ordinates (higher value indicates reddish and lower value indicates greenish tint) and, b* represents blue to yellow co-ordinates (higher value means bluish and lower value means yellowish tints) [5, 18]. Additionally, CIE also standardized how color variations could be measured from CIELAB values, where color differences between two subjects could be calculated by using the ‘Euclidian’ color difference equation, commonly referred as ‘ΔE’ [20, 23].

Therefore, the current study aimed to capture images of pigmented prosthetic silicone elastomers placed within 2 windowed and 2 windowless clinics at different clinically relevant lighting conditions at a particular time of a day (noon) to 1) evaluate the CIELAB color variations present in facial prosthetic specimens among different clinical settings, and 2) determine the amount of color variations that can be digitally corrected by using different calibration methods (CWBC and PPWBC).

It was hypothesized that, 1) the CIELAB values within captured images will not be significantly affected by the lighting variations present in different clinics and, 2) the choice of photo calibration method will not be significantly affected by the clinical environments where they are being photographed.

## Materials and methods

### Sample size calculation and data collection

Based on a medium effect size of 0.25 (Cohen’s d) [24]; α = .05 and power of study = .95, 324 measurements were indicated. After considering human or environment-generated errors [9] and possibilities of instrumental metamerism *(i*.*e*., *when two colors matched under certain conditions greatly differ to one another)* [25] that can occur due to camera calibrations, lens selection, illumination sources and color temperatures [26–28]; an additional 33% measurements were included, therefore, 432 measurements were taken from 6 groups (n = 72) of photographs: photo box, 2 windowed clinics, 2 windowless clinics, and one Spectrophotometer (control).

### Template design

Templates were designed in a computer-aided design software (FreeCAD; AutoDesk, San Rafael, California, USA) with the dimensions of 40 × 20 × 8 mm^3^ [29]. A desktop 3D printer (Ender-3; Creality, Shenzhen, China) was used to print the templates using thermoplastic polyurethane (TPU) filament (1.75 mm).

### Mold preparation

The templates were coated in petroleum jelly (separating media) and invested into a metallic flask filled with semi-set greenstone (Saint Gobain; ACME MD Supply, Pulau Pinang, Malaysia) which helped to create a mold for the silicone polymerization after the templates were removed from the set cast.

### Preparation of silicone blocks

A-103 maxillofacial prosthetic silicone elastomer [30, 31] (A-103; Factor II, Lakeside, USA) [32] was used as material core and 2 functional intrinsic skin pigments Blush and Honey (FI-SK13, FI-SK09; Factor II, Lakeside, AZ, USA) were incorporated individually to color the unpolymerized silicone. A digital weight machine (Analytical Digital Balance BSA423S; Sartorius, Goettingen, Germany) was used to measure the pigments (2% by weight) [29] and A-103 silicone elastomer with a ratio of 10:1 (according to the manufacturer instruction). The silicone and pigments were first hand mixed [33] and then processed through a vacuum mixer (MIXYVAC T; Manfredi, San Secondo di Pinerolo, Italy) to allow for even dispersion of pigments within the silicone matrix [34, 35]. Afterwards, the pigmented silicone was packed and polymerized following conventional standard protocol [29] and marked from where the measurements were taken.

### Image capture procedure

#### Photographic standardization

All the photographs were taken on a sunny midday using a Single-Lens Reflex (SLR) digital camera (NIKON D610; Nikon, Tokyo, Japan) with a 105 mm macro lens. The camera was standardized manually to flash disabled [12], ISO 800, aperture 3.0/f, 1/160 shutter speed, color temperature was 5880 K. A smartphone-based lux meter (Lux light meter; DoggoApps, Moscow, Russia) was used to monitor the luminescence *(i*.*e*., *continuous discharge of light by a substance which is caused not because of heat but because of ongoing chemical reaction and electrical energies)*. The camera was standardized at a fixed 80-degree angle to face the silicone blocks on a tripod with a height of 120 cm. Moreover, to avoid any unwanted light reflections [36, 37], a matte black platform was used, which was serve as specimen background and was positioned 45 cm above the ground.

To answer research question 1, pigmented silicone blocks were photographed under 5 different environmental conditions, namely photo box (to simulate intra-oral condition) [38], two identical windowed (windowed clinic 1 and 2) and windowless clinics (windowless clinic 1 and 2). A miniature 3 walled enclosure called Puluz mini studio photo box (Puluz mini studio; Puluz, Shenzhen, China) was used with the luminescence of 80–90 lux. Afterwards, the samples were again photographed in the center of two fully lit windowed and windowless prosthodontic clinics with the luminescence of 370–430 and 450–470 lux, respectively. Of note, all photographs were taken under the light available within the clinics, no external light influencers (i.e., ring lights, LED lights) or diffusers, or polarizing filters were used during each photographic session.

To answer research question 2, the images were calibrated by using different color calibration methods namely, a) no color calibration images (raw image), b) CWBC by using gray reference card [8, 10], c) PPWBC by using gray card and Macbeth color chart [8, 39]. CWBC and PPWBC procedures are described as follow:

- ***CWBC by using gray card*:**

An 18% professional neutral gray color card (18% Gray Card; Anwenk, Shenzhen, China) was used to calibrate the camera for each environmental condition prior to image capture [5, 11]. The camera auto white balance was set to default to eliminate possibilities of altered brightness and gray tone within the images as no external lights were used during photography [40]. An 18% gray card was placed within a picture frame and the captured image was used to calibrate the camera [1].

- ***PPWBC by using gray card*:**

The white balance of the camera was set to default and an 18% gray card was placed along with the pigmented silicone blocks on a black background in order to establish a reference white balance [1, 5, 39]. The images were captured within each environmental condition and later corrected on a computer.

- ***PPWBC by using Macbeth color chart*:**

A professional Macbeth color chart (Spyderchekr 24; Datacolor, Trenton, NJ, USA) [5, 39, 41, 42] was positioned in the place of the 18% gray card and the images were repeated within each environmental condition following the same procedure. However, photographs with Macbeth color chart were not done as the photo box cast uneven shadows onto the color charts and could potentially skew the data.

**Fig 1:**
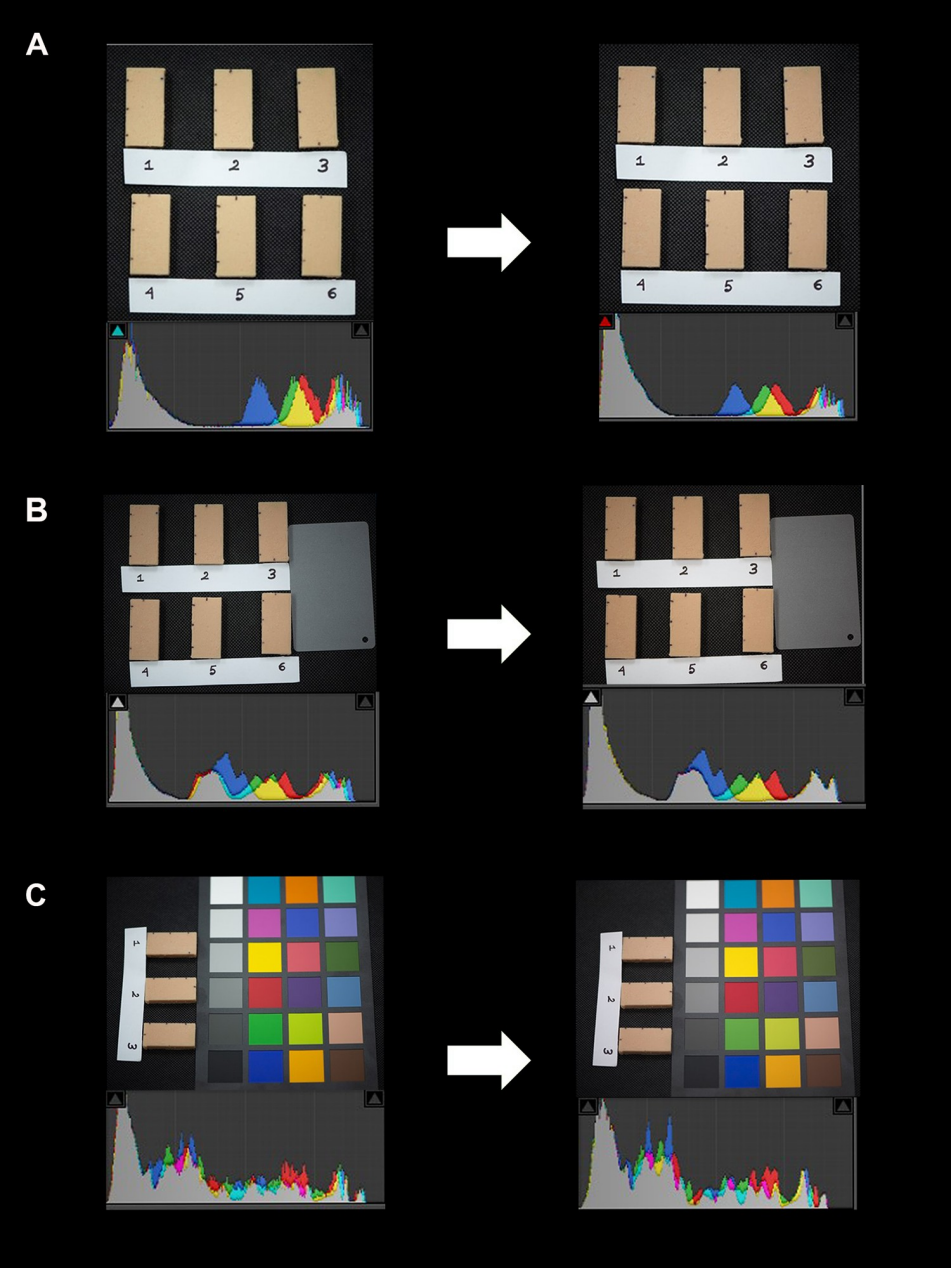
Before after color differences of prosthetic silicone blocks for A) camera calibration, B) PPWBC using gray card and C) PPWBC using color chart.

**Fig 2:**
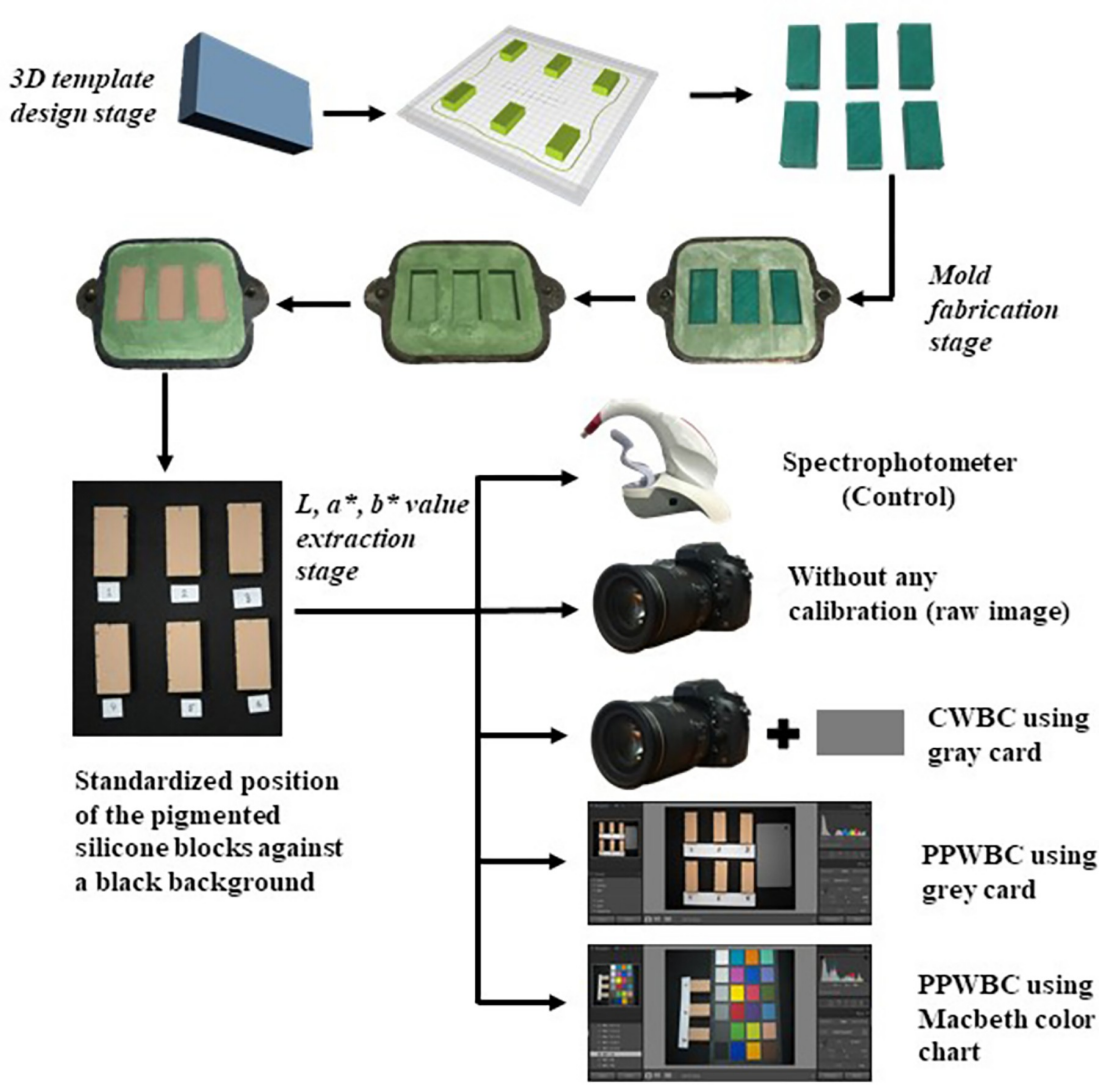
Workflow summary of the current study.

- *Computerized white balance calibration and L*, a*, b* value extraction procedure:

The captured images were exported to a photo editing software (Adobe Photoshop lightroom CC; Adobe, San Jose, California, USA) using NEF format, where the white balance was calibrated according to the 18% gray card and Macbeth color chart [5]. Afterwards, L, a*, b* values were extracted from the pigmented silicone block images **(see Fig 1 and** S1 **to S3 Tables)**. A summary of the entire workflow has been presented in **Fig 2**.

### Color difference (ΔE) calculation

The following formula was used to evaluate the color difference:

$$\Delta E=\surd ({\Delta L)}^{2}+{\left(\Delta a\right)}^{2}+{\left(\Delta b\right)}^{2}$$

Here, ΔL means lightness/darkness, [ΔL = (L* value sample–L* value standard); Δa means red/green axis, [Δa = (a* value sample–a* value standard); Δb means yellow/blue axis [Δb = (b* value sample–b* value standard) [35]

### Statistical analyses

Analyses were carried out using the Statistical Package for Social Sciences software (SPSS, Version 26.0; IBM, Armonk, NY, USA). Normality of data were tested using Shapiro Wilk test and L, a* and b* values were independently analyzed by Kruskal-Wallis one-way test. Additionally, Post-hoc pairwise analysis was carried out using Dunn’s Test reporting standardized test statistics (t-stat)

## Results

### Results without white-balance calibration

When L, a* b* values obtained from the photographs without any white balance corrections (raw images) were compared against spectrophotometric values, there were highly significant differences (p < 0.001) observed for all the ambient lighting conditions ***(Table 1)***, which indicates that the overall CIELAB values were affected by all environmental lighting variations, therefore, the null hypothesis for research aim 1) is rejected. Additionally, post-hoc analysis (Dunn’s test) revealed no significant differences thereby partially accepting the null hypothesis of research aim 2 for the following conditions:

- L values obtained from 1) spectrophotometer *vs*. windowed clinic 2 (t-stat = .662, p = 0.508); 2) photo box *vs*. windowed clinic 1 (t-stat = -1.030, p = 0.303); 3) photo box *vs*. windowless clinic 1 (t-stat = -1.131, p = 0.258); and 4) windowless clinic 1 *vs*. windowed clinic 1 (t-stat = .101, p = 0.919)
- a* values obtained from 1) spectrophotometer *vs*. windowless clinic 1 (t-stat = -1.492, p = 0.136); 2) windowless clinic 1 *vs*. windowless clinic 2 (t-stat = -1.657, p = 0.098); and 3) windowed clinic 1 *vs*. windowed clinic 2 (t-stat = 1.259, p = 0.208)
- b* values obtained from 1) spectrophotometer *vs*. photo box (t-stat = .019, p = 0.985); and 2) windowless clinic 1 *vs*. windowed clinic 2 (t-stat = -1.639, p = 0.101)

**Table 1:** Comparison of L, a* and b* values for images without any white balance correction (raw images).

**L**			
	***Median (IQR)***	***χ***^***2***^ ***(df)***	P* ^***a***^
Spectrophotometer (control)	62.45 (7.8)	226.691(5)	p = 4.682 × 10^−62^
Photo box	71.50 (9.8)		
Windowless clinic 1	73.00 (10.8)		
Windowless clinic 2	57.00 (15.0)		
Windowed clinic 1	72.00 (7.0)		
Windowed clinic 2	61.00 (12.8)		
b*			
	***Median (IQR)***	***χ***^***2***^ ***(df)***	p* ^***b***^
Spectrophotometer (control)	7.00 (5.1)	260.727 (5)	P = 1.267 × 10^−17^
Photo box	20.50 (3.0)		
Windowless clinic 1	7.00 (2.0)		
Windowless clinic 2	8.00 (4.0)		
Windowed clinic 1	10.00 (2.0)		
Windowed clinic 2	9.00 (3.0)		
b*			
	***Median (IQR)***	***χ***^***2***^ ***(df)***	P* ^***c***^
Spectrophotometer (control)	43.20 (10.7)	335.766 (5)	p = 1.201 × 10^−19^
Photo box	42.00 (2.0)		
Windowless clinic 1	22.00 (4.0)		
Windowless clinic 2	21.00 (2.8)		
Windowed clinic 1	27.00 (5.8)		
Windowed clinic 2	24.00 (3.8)		

*Significant < 0.05; χ^2^ = Chi-square statistics; df = degree of freedom, IQR = Interquartile Range

*Kruskal-Wallis one-way test: Parametric assumption not met. Shapiro- Wilk test significant (p < 0.05)

^a^ Post-hoc analysis (Dunn’s test) for L: Null hypothesis rejected (p = 0.0 × 10^0^ < 0.001). Spectrophotometer *vs* all other lighting conditions showed highly significant differences (p < 0.001) except windowed clinic 2 (p = 0.508). No significant difference was observed for photo box *vs* windowed clinic 1 (p = 0.303), photo box *vs* windowless clinic 1 (p = 0.258), and windowless clinic 1 *vs* windowed clinic 1 (p = 0.919)

^b^ Post-hoc analysis (Dunn’s test) for a*: Null hypothesis rejected (p < 0.001). Spectrophotometer *vs* all other lighting conditions showed highly significant differences (p < 0.001) except windowless clinic 1 (p = 0.136). No significant differences observed for windowless clinic 1 *vs* windowless clinic 2 (p = 0.098) and windowed clinic 1 *vs* windowed clinic 2 (p = 0.208)

^c^ Post-hoc analysis (Dunn’s test) for b*: Null hypothesis rejected (p < 0.001). Spectrophotometer *vs* all other lighting conditions showed highly significant differences (p < 0.001) except photo box (p = 0.985). No significant difference observed for windowless clinic 1 *vs* windowed clinic 2(p = 0.101)

### Results for CWBC

When L, a*, b* values obtained from the camera calibrated images were evaluated and compared against the spectrophotometric values, they exhibited highly significant differences (p < 0.001), thereby rejecting the null hypothesis 1 ***(Table 2)*.** On the contrary, Post-hoc analysis (Dunn’s test) revealed no significant differences, thus partially accepting the null hypothesis for research aim 2 for following conditions:

- L values obtained from 1) spectrophotometer *vs*. windowless clinic 2 (t-stat = -1.673, p = 0.094); 2) photo box *vs*. windowed clinic 2 (t-stat = -1.256, p = 0.209); 3) photo box *vs*. windowless clinic 1 (t-stat = -1.686, p = 0.092); 4) windowed clinic 2 *vs*. windowless clinic 1 (t-stat = .430, p = 0.667); 5) windowed clinic 1 *vs*. windowed clinic 2 (t-stat = .827, p = 0.408) and 6) windowless clinic 1 *vs*. windowed clinic 1 (t-stat = -.398; p = 0.691)
- a* values obtained from 1) windowless clinic 1 *vs*. windowless clinic 2 (t-stat = -.459, p = 0.646); and 2) windowed clinic 1 *vs*. windowed clinic 2 (t-stat = 1.526, p = 0.127)
- b* values obtained from 1) windowed clinic 2 *vs*. windowless clinic 2 (t-stat = .435, p = 0.663); 2) windowed clinic 1 *vs*. windowed clinic 2 (t-stat = .701; p = 0.483); and 3) windowless clinic 2 *vs*. windowed clinic 1 (t-stat = .266; p = 0.790)

**Table 2:** Comparison of L, a* and b* values for images with camera white balance correction.

**L**			
	***Median (IQR)***	***χ***^***2***^ ***(df)***	**p** ^*^ ^***a***^
Spectrophotometer (control)	62.45 (7.80)	120.875 (5)	p = 2.474 × 10^−28^
Photo box	54.00 (10.75)		
Windowless clinic 1	56.00 (12.00)		
Windowless clinic 2	65.00 (10.00)		
Windowed clinic 1	56.00 (15.75)		
Windowed clinic 2	55.50 (12.75)		
a*			
	***Median (IQR)***	***χ***^***2***^ ***(df)***	**p** ^*^ ^***b***^
Spectrophotometer (control)	7.00 (5.10)	239.923 (5)	p *=* 8.683 × 10^−20^
Photo box	22.00 (2.0)		
Windowless clinic 1	7.50 (4.0)		
Windowless clinic 2	8.00 (4.00)		
Windowed clinic 1	10.00 (2.0)		
Windowed clinic 2	9.00 (3.0)		
b*			
	***Median (IQR)***	***χ***^***2***^ ***(df)***	**p** ^*^ ^***c***^
Spectrophotometer (control)	43.20 (10.68)	320.036 (5)	p *=* 9.412 × 10^−18^
Photo box	33.50 (5.75)		
Windowless clinic 1	20.00 (3.00)		
Windowless clinic 2	22.00 (3.00)		
Windowed clinic 1	23.00 (3.00)		
Windowed clinic 2	22.00 (3.00)		

*Significant < 0.05; χ^2^ = Chi-square statistics; df = degree of freedom, IQR = Interquartile Range

*Kruskal-Wallis one-way test: Parametric assumption not met. Shapiro- Wilk test significant (p < 0.05)

^a^ Post-hoc analysis (Dunn’s test) for L: Null hypothesis rejected (p < 0.001). Spectrophotometer *vs* all other lighting conditions showed highly significant differences (p < 0.001) except windowless clinic 2 (p = 0.094). No significant differences observed for photo box *vs* windowed clinic 2 (p = 0.209), photo box *vs* windowless clinic 1 (p = 0.092), windowless clinic 1 *vs* windowed clinic 2 (p = 0.667), widowed clinic 1 *vs* windowed clinic 2 (p = 0.408), windowless clinic 1 *vs* windowed clinic 1 (p = 0.691)

^b^ Post-hoc analysis (Dunn’s test) for a*: Null hypothesis rejected (p < 0.001). Spectrophotometer *vs* all other lighting conditions showed highly significant differences (p < 0.001). No significant differences observed for windowless clinic 1 *vs* windowless clinic 2 (p = 0.646), windowed clinic 1 *vs* windowed clinic 2 (p = 0.127)

^c^ Post-hoc analysis (Dunn’s test) for b*: Null hypothesis rejected (p < 0.001). Spectrophotometer *vs* all other lighting conditions showed highly significant differences (p < 0.001). No significant differences observed for windowed clinic 2 *vs* windowless clinic 2 (p = 0.663), windowed clinic 1 *vs* windowed clinic 2 (p = 0.483), windowless clinic 2 *vs* windowed clinic 1 (p = 0.790)

### PPWBC using gray card

Post processing white balance correction using gray card demonstrated highly significant differences (p < 0.001) for L, a*, b* values, thereby rejecting null hypothesis 1 ***(Table 3)***. However, Post-hoc analysis (Dunn’s test) revealed no significant differences, thereby partially accepting the null hypothesis for research aim 2 for the following conditions:

- L values obtained from 1) spectrophotometer *vs*. windowed clinic 2 (t-stat = 1.325, p = 0.185), 2) photo box *vs*. windowless clinic 1 (t-stat = -1.234, p = 0.217) and 3) windowless clinic 1 *vs*. windowless clinic 2 (t-stat = -1.533, p = 0.125)
- a* values obtained from 1) windowless clinic 1 *vs*. windowless clinic 2 (t-stat = -.659, p = 0.510); and 2) windowed clinic 1 *vs*. windowed clinic 2 (t-stat = -.209, p = 0.834)
- b* values obtained from 1) photo box *vs*. windowless clinic 1 (t-stat = -1.029, p = 0.303); 2) windowless clinic 1 *vs*. windowless clinic 2 (t-stat = -1.355, p = 0.175); and 3) windowed clinic 1 *vs*. windowed clinic 2 (t-stat = 1.304, p = 0.192)

**Table 3:** Comparison of L, a* and b* values for images with post processing white balance correction using gray card.

**L**			
	***Median (IQR)***	***χ***^***2***^ ***(df)***	**p** ^*^ ^***a***^
Spectrophotometer (control)	62.45 (7.80)	156.875 (5)	p = 4.346 × 10^−45^
Photo box	50.00 (15.75)		
Windowless clinic 1	54.00 (12.75)		
Windowless clinic 2	57.00 (14.00)		
Windowed clinic 1	72.00 (14.00)		
Windowed clinic 2	60.50 (13.75)		
a*			
	***Median (IQR)***	***χ***^***2***^ ***(df)***	**p** ^*^ ^***b***^
Spectrophotometer (control)	7.00 (5.10)	281.707 (5)	p = 3.076 × 10^−11^
Photo box	15.00 (3.00)		
Windowless clinic 1	11.00 (3.75)		
Windowless clinic 2	11.00 (3.00)		
Windowed clinic 1	13.00 (2.00)		
Windowed clinic 2	13.00 (2.00)		
b*			
	***Median (IQR)***	***χ***^***2***^ ***(df)***	**p** ^*^ ^***c***^
Spectrophotometer (control)	43.20 (10.68)	235.572 (5)	p = 4.332 × 10^−14^
Photo box	22.00 (4.75)		
Windowless clinic 1	23.00 (3.00)		
Windowless clinic 2	25.00 (4.00)		
Windowed clinic 1	26.50 (4.00)		
Windowed clinic 2	26.00 (4.00)		

*Significant < 0.05; χ^2^ = Chi-square statistics; df = degree of freedom, IQR = Interquartile Range

*Kruskal-Wallis one-way test: Parametric assumption not met. Shapiro- Wilk test significant (p < 0.05)

^a^ Post-hoc analysis (Dunn’s test) for L: Null hypothesis rejected (p < 0.001). Spectrophotometer *vs* all other lighting conditions showed highly significant differences (p < 0.001) except windowed clinic 2 (p = 0.185). No significant differences observed for photo box *vs* windowless clinic 1 (p = 0.217), windowless clinic 1 *vs* windowless clinic 2 (p = 0.125)

^b^ Post -hoc analysis (Dunn’s test) for a*: Null hypothesis rejected (p < 0.001). Spectrophotometer *vs* all other lighting conditions showed highly significant differences (p < 0.001). No significant differences observed for windowless clinic 1 *vs* windowless clinic 2 (p = 0.510), windowed clinic 1 *vs* windowed clinic 2 (p = 0.834).

^c^ Post -hoc analysis (Dunn’s test) for b*: Null hypothesis rejected (p < 0.001). Spectrophotometer *vs* all other lighting conditions showed highly significant differences (p < 0.001). No significant differences observed for photo box *vs* windowless clinic 1 (p = 0.303), windowless clinic 1 *vs* windowless clinic 2 (p = 0.175), windowed clinic 1 *vs* windowed clinic 2 (p = 0.192)

### PPWBC using Macbeth color chart

Post processing white balance correction using Macbeth color chart demonstrated no significant differences (p > 0.05) for a* values with significant differences for L and b* values, therefore, partially rejecting null hypothesis 1 ***(Table 4)***. Additionally, post-hoc analysis (Dunn’s test) revealed no significant differences, thereby partially accepting the null hypothesis, for the following conditions:

- L values obtained from 1) spectrophotometer *vs*. windowed clinic 1 (t-stat = -.104, p = 0.917); 2) spectrophotometer *vs*. windowed clinic 2 (t-stat = -1.021, p = 0.307); 3) spectrophotometer *vs*. windowless clinic 2 (t-stat = -1.681, p = 0.093); 4) windowed clinic 1 *vs*. windowed clinic 2 (t-stat = -.917, p = 0.359); 5) windowed clinic 1 *vs*. windowless clinic 2 (t-stat = 1.578, p = 0.115); 6) windowed clinic 2 *vs*. windowless clinic 2 (t-stat = .661, p = 0.509); 7) windowless clinic 1 *vs*. windowless clinic 2 (t-stat = 1.879, p = 0.060)
- b* values obtained from 1) windowless clinic 1 *vs*. windowless clinic 2 (t-stat = .625, p = 0.532); and 2) windowed clinic 1 *vs*. windowed clinic 2 (t-stat = .058, p = 0.953)

**Table 4:** Comparison of L, a* and b* values for images with post processing white balance correction using Macbeth color chart.

**L**			
	***Median (IQR)***	***χ***^***2***^ ***(df)***	p^*^ ^***a***^
Spectrophotometer (control)	62.45 (7.80)	16.904 (4)	p = 0.000
Windowless clinic 1	64.45 (9.60)		
Windowless clinic 2	63.00 (7.38)		
Windowed clinic 1	62.00 (11.15)		
Windowed clinic 2	62.70 (13.25)		
a*			
	***Median (IQR)***	***χ***^***2***^ ***(df)***	P* ^***b***^
Spectrophotometer (control)	7.00 (5.10)	8.933 (4)	p = 0.015
Windowless clinic 1	6.75 (4.15)		
Windowless clinic 2	6.60 (3.43)		
Windowed clinic 1	6.80 (3.10)		
Windowed clinic 2	6.80 (3.30)		
b*			
	***Median (IQR)***	***χ***^***2***^ ***(df)***	P* ^***c***^
Spectrophotometer (control)	43.20 (10.68)	205.699 (4)	p = 8.376 × 10^−16^
Windowless clinic 1	20.05 (3.58)		
Windowless clinic 2	19.80 (3.20)		
Windowed clinic 1	21.70 (3.38)		
Windowed clinic 2	21.85 (3.25)		

*Significant < 0.05; χ^2^ = Chi-square statistics; df = degree of freedom, IQR = Interquartile Range

*Kruskal-Wallis one-way test: Parametric assumption not met. Shapiro- Wilk test significant (p < 0.05)

^a^ Post-hoc analysis (Dunn’s test) for L: Null hypothesis rejected (p = 0.002). Spectrophotometer Vs all other lighting conditions showed no significant differences (p > 0.05) except windowless clinic 1 (p < 0.001). Additionally, no significant differences observed for windowed clinic 1 *vs* windowed clinic 2 (p = 0.359), windowed clinic 1 *vs* windowless clinic 2 (p = 0.115), windowed clinic 2 *vs* windowless clinic 2 (p = 0.509), windowless clinic 1 *vs* windowless clinic 2 (p = 0.060)

^b^ Post -hoc analysis (Dunn’s test) for a*: Null hypothesis accepted (p = 0.063)

^c^ Post -hoc analysis (Dunn’s test) for b*: Null hypothesis rejected (p < 0.001). Spectrophotometer *vs* all other lighting conditions showed highly significant differences (p < 0.001). No significant differences observed between windowless clinic 1 *vs* windowless clinic 2 (p = 0.532) and windowed clinic 1 *vs* windowed clinic 2 (p = 0.953)

### Color differences (ΔE) between the techniques

CWBC demonstrated no significant differences in LAB values across windowed clinics while PPWBC using gray card showed no significant differences in LAB across windowless clinics. In contrast, PPWBC using Macbeth color chart produced no significant LAB differences across both windowed groups and windowless groups. Details of significance and trends have been tabulated in **S4 Table.**

Upon ΔE evaluation **(see S5 Table)**, photo box produced the lowest ΔE value without white balance correction and camera calibration (ΔE = 16.83 and 19.68, respectively). Windowless clinics (ΔE = 20.23) and windowed clinics (ΔE = 20.15) produced similar ΔE upon camera calibration. Though both gray cards in post-processing produced the lowest values for both windowless (ΔE = 19.57) and windowed (ΔE = 17.64) clinics while color chart produced favorable values only for the windowed clinics (ΔE = 19.15). Gray card and Macbeth color chart had the most color differences in both windowless (ΔE = 10.87) and windowed (ΔE = 9.59) clinics. Camera calibration and post processing gray card calibration had lesser differences in windowless clinics (ΔE = 6.46) as opposed to windowed clinics (ΔE = 12.02)

## Discussion

The study aimed to evaluate 1) the amount of color variations presents within clinical photographs of maxillofacial prosthetic silicone elastomeric specimen when captured under different clinically relevant ambient lighting conditions, and 2) whether white balance calibration (WBC) methods were able to mitigate such lighting variations.

The study findings suggest that L, a*, b* values of pigmented maxillofacial silicone elastomer within each clinical scenario were significantly affected by the ambient lighting variations. Likely explanations may include that the spectrophotometer (Vita Easyshade Advance 4.0; VITA Zahnfabrik, Bad Säckingen, Baden-Württemberg, Germany) which was used as control is primarily designed for dental applications (i.e., evaluation of tooth shade), therefore it is factory-calibrated to exclusively highlight a variety of tooth dependent factors (i.e., tooth anatomy, translucency and thickness of enamel and dentin) [19]. Additionally, the current study relied on natural daylight to mimic a real dental scenario at its peak operating hours [7, 8]. The intensity and direction of sunlight changes during a day, hence there remains a chance that the bluish tints within the photographs are drastically affected by the ambient lights because blue color from the sunlight disperses/scatters the most and travels in shorter and smaller waves [38].

A study [25] documented that, studio images of a tooth shade guide with a standardized environment (i.e., two light flashes with one soft box lamp, 600 W, respectively) produced significantly different (p < 0.01) values (L and RGB) when compared with daylight images. Therefore, investigators concurred that instrument-based variables such as camera aperture (*which dictates the amount of light from the environment accessing the image sensor*), shutter speed (*i*.*e*., *the speed at which the shutter closes*), color temperature (*i*.*e*., *a numerical system measured in Kelvin that measures the color based on its warm/reddish to cool/bluish spectrum*), resolution (*i*.*e*., *number of pixels within an image*) and white balance are need to be modified based on the specific environment in which the photographs are being taken to avoid instrumental metamerism and generate color accurate images [28, 43].

The calibration tools were effective in standardizing the ambient light *among* similar clinics (windowed *or* windowless) while proving to be generally ineffective when compared *between* the clinics (windowed *and* windowless) **(See S4 Table)**.This is in agreement with other authors investigating properties of color in the dental space [1, 5, 8, 10]. A possible explanation is that correction tools are unable to solve discrepancies within a greater range of color intensities present across windowed and windowless environments. The ultraviolet light entering the clinics alter the chemical bonds of the colorants present within the gray card and introduce color shifts otherwise not seen in a windowless setup [44]. In said instances, a color temperature conversion filter or diffuser have been suggested to remove unwanted reflections and glares [11, 39, 45] by remapping the color values based on the ambient color temperature in real time and adjusting to the temperature variations seen throughout the day; a moderately overcast sky has a color temperature of 6500–8000 K whereas the temperature in a closed room/studio photography varies between 2500–3500 K.

It is argued that standardization alone may not be enough, thus professional color calibration tools such as gray card or Macbeth color chart need to be introduced in the photo sessions [1, 46, 47]. Current findings partially agree with previous reports [46–48], where gray reference card and Macbeth color charts were able to neutralize the image brightness brought upon by ambient lighting variations and provide more standardized (and therefore realistic) color values when compared to normal images (ΔE = 3.4) [1]. Moreover, windowless clinics are illuminated by fluorescent lighting which emits a bluish tint of light onto photographs. In that case, current findings as well as the past reports suggest that, gray cards were particularly effective in neutralizing those bluish tint within windowless clinics [38, 49].

PPWBC using Macbeth color chart produced consistently favorable results across the different clinics by reliably correcting dynamic lighting conditions [50] for the silicone elastomer. This might be because the reflectance spectrum and color co-ordinates of the color patches are carefully formulated which help to avoid gamut clipping (*i*.*e*., *when different colors within the captured photograph turn out similar after printing*) under various lighting conditions, whereas gray card is typically used to balance the natural colors where the spectral reflectance is uniform. In non-uniform reflectance, they might reflect more light which may hamper the camera’s sensitivity of accurate color balance. To avoid such issues during outdoor photography, a Macbeth color chart would be considered as more viable than gray cards [44]. Nevertheless, arguments against such methods of white balance correction mention alteration of color values as a collateral effect while reducing environmental noises and unwanted color casts [51].

In order to mimic photographs taken within an intraoral environment; the current study used an unlit photo box. The investigation found a* (red/green axis) and b* (yellow/blue axis) were higher for all clinical scenarios; possibly because the enclosure reflected excess natural light which consequently increases the color contrast [8]. In practice, this will create unrealistic red colorcasts for the gingiva images and will produce yellowness for the teeth [38]. But the situation was favorable for the current study, when PPWBC was done using a gray card and the photo box was placed within a windowless clinic [8, 10].

Despite the variations, human perceptibility [52] was found to clinically accept a color variation of up to 3.7 with higher values yielding visually unacceptable results for dental tissue [1, 26]. It should however be noted that research on natural skin color yielded varying ΔE values ranging from 11.62 to 36.91 for no white balance (raw images) and 9.77 to 15.13 after white balance calibration [5, 39, 53, 54]. Results from the current *in vitro* study were primarily different because living skin tissue showed higher variations based on its patho-physiologic distribution and weather exposure otherwise absent in prosthetic silicone material [5]. As there are limited studies on color space, digitization and property analyses in maxillofacial prosthetic silicone, this may further limit the conclusiveness and detailed explanations for the current findings [33, 55–57].

Evidently, digital dentistry has adopted non-contact color measuring devices such as professional digital camera, intraoral cameras [58, 59] and smartphone cameras [1, 10] to ease procedures of tooth color analysis within the dental clinics. Nowadays, to generate 3-dimensional virtual models, an intraoral scanner is mostly used. However these scanners do not generally provide color information [60]. Since 2016, Machine Learning (ML) and Artificial Intelligence (AI) have been digitally implemented to aid objective shade analyses [61, 62]. A study [63] had reported minimal color variations when the investigators incorporated ML with tooth shade matching in order to mitigate ambient light variations. Similar implementations on prosthetic silicone are lacking as of now and can be topics of future exploration.

Finally, the data was limited by the *in vitro* nature of the study design and geographic variations since only dental clinics within a single center of a tropical country (Malaysia) were considered. Future *in vivo* studies on color variation in facial prostheses are recommended that can also take into account the influence of human sebum and weathering on color variations. Multi-center evaluations across several climates should be carried out to establish ΔE values that can be considered clinically acceptable for maxillofacial prostheses. While the current study was limited by software-based luminescence indicators, future investigations are encouraged to use professional hardware to record dynamic lighting variations across the clinics. Further studies can be carried out to evaluate the influence of clinic architecture (ex. number of windows and interior decor) on the color variations within dental and prosthetic photography.

## Conclusion

Based on the current *in vitro* report, clinics exposed to natural ambient light through windows can drastically influence photographs taken of facial prosthetic silicone elastomer. The choice of color calibration method should be case-specific to the clinics where the photographs are being taken.

## Supporting information

S1 Table L values from the pigmented silicone samples. (DOCX)

S2 Table a* values from the pigmented silicone samples. (DOCX)

S3 Table b* values from the pigmented silicone samples. (DOCX)

S4 Table Dunn’s test results at different environmental conditions. (DOCX)

S5 Table Color differences (ΔE) produced between different methods of color calibration in relevant clinical lighting scenarios. (DOCX)
