# Supplementary material for: Color variations during digital imaging of facial prostheses subjected to unfiltered ambient light and image calibration techniques within dental clinics: An in vitro analysis
Source: PLoS One. 2022 Aug 29;17(8):e0273029. doi: 10.1371/journal.pone.0273029 (PMC9423681; doi:10.1371/journal.pone.0273029)
Supplement: S1 Table — (DOCX) [file pone.0273029.s001.docx]

# S1 Table. L values from the pigmented silicone samples

| **Sample measurement** | **Spectro-photometer** | **Images without any white balance corrections  (Raw images)** | | | | | **CWBC** | | | | | **PPWBC using gray card** | | | | | **PPWBC using Macbeth color chart** | | | |
| --- | --- | --- | --- | --- | --- | --- | --- | --- | --- | --- | --- | --- | --- | --- | --- | --- | --- | --- | --- | --- |
|  |  | Photo box | Windowless clinic 1 | Windowless clinic 2 | Windowed clinic 1 | Windowed clinic 2 | Photo box | Windowless clinic 1 | Windowless clinic 2 | Windowed clinic 1 | Windowed clinic 2 | Photo box | Windowless clinic 1 | Windowless clinic 2 | Windowed clinic 1 | Windowed clinic 2 | Windowless clinic 1 | Windowless clinic 2 | Windowed clinic 1 | Windowed clinic 2 |
| 1a | 65.2 | 69 | 66 | 52 | 67 | 61 | 53 | 60 | 67 | 55 | 60 | 50 | 64 | 55 | 70 | 61 | 71.4 | 72.3 | 71.3 | 71.8 |
| 1b | 64.6 | 73 | 71 | 57 | 72 | 62 | 59 | 60 | 69 | 53 | 61 | 50 | 66 | 58 | 78 | 64 | 70.5 | 67.4 | 70.8 | 69.9 |
| 1c | 64.4 | 75 | 71 | 58 | 73 | 64 | 57 | 61 | 69 | 60 | 62 | 53 | 64 | 59 | 76 | 67 | 67.9 | 67.8 | 66.4 | 69.3 |
| 2a | 66.4 | 70 | 75 | 58 | 72 | 68 | 50 | 60 | 68 | 54 | 65 | 53 | 65 | 63 | 72 | 68 | 71.1 | 71.5 | 68.3 | 69.0 |
| 2b | 64.9 | 73 | 78 | 63 | 74 | 72 | 46 | 62 | 71 | 65 | 67 | 58 | 68 | 63 | 78 | 70 | 68.8 | 71.5 | 72.5 | 73.2 |
| 2c | 67.2 | 82 | 78 | 64 | 78 | 75 | 55 | 68 | 74 | 62 | 67 | 64 | 67 | 64 | 78 | 76 | 67.7 | 67.9 | 72.1 | 75.7 |
| 3a | 65.8 | 64 | 69 | 63 | 70 | 71 | 55 | 57 | 68 | 63 | 62 | 57 | 65 | 66 | 67 | 73 | 64.3 | 62.7 | 64.8 | 65.8 |
| 3b | 65.0 | 74 | 74 | 60 | 69 | 69 | 51 | 59 | 64 | 58 | 59 | 53 | 64 | 63 | 74 | 67 | 66.4 | 65.4 | 67.4 | 70.3 |
| 3c | 64.7 | 76 | 78 | 61 | 78 | 67 | 47 | 61 | 64 | 57 | 61 | 48 | 64 | 57 | 69 | 70 | 66.8 | 67.0 | 68.0 | 69.4 |
| 4a | 66.0 | 81 | 77 | 64 | 74 | 64 | 61 | 67 | 72 | 65 | 64 | 63 | 60 | 66 | 80 | 65 | 74.9 | 70.9 | 70.2 | 71.3 |
| 4b | 66.0 | 82 | 75 | 60 | 70 | 62 | 64 | 67 | 68 | 64 | 59 | 62 | 60 | 59 | 77 | 66 | 75.8 | 71.3 | 68.7 | 69.9 |
| 4c | 64.1 | 81 | 74 | 60 | 75 | 61 | 57 | 67 | 69 | 64 | 51 | 60 | 56 | 63 | 83 | 64 | 72.5 | 69.1 | 68.8 | 68.6 |
| 5a | 66.6 | 82 | 83 | 70 | 85 | 75 | 61 | 71 | 75 | 69 | 68 | 64 | 65 | 73 | 86 | 74 | 77.9 | 72.9 | 72.9 | 73.5 |
| 5b | 68.5 | 83 | 81 | 68 | 82 | 70 | 59 | 72 | 73 | 69 | 65 | 66 | 58 | 71 | 85 | 74 | 75.8 | 69.8 | 69.1 | 71.4 |
| 5c | 66.7 | 82 | 78 | 67 | 75 | 69 | 58 | 71 | 71 | 69 | 64 | 69 | 56 | 62 | 86 | 68 | 73.8 | 68.6 | 68.5 | 66.5 |
| 6a | 66.0 | 82 | 78 | 67 | 76 | 71 | 55 | 64 | 70 | 67 | 63 | 62 | 62 | 68 | 80 | 74 | 72.9 | 67.6 | 68.3 | 69.8 |
| 6b | 66.9 | 81 | 75 | 67 | 79 | 67 | 54 | 63 | 69 | 67 | 61 | 62 | 60 | 70 | 81 | 68 | 72.7 | 66.9 | 67.2 | 67.8 |
| 6c | 66.7 | 81 | 74 | 65 | 68 | 66 | 55 | 62 | 66 | 64 | 57 | 63 | 58 | 68 | 79 | 67 | 71.5 | 64.6 | 65.9 | 64.9 |
| 7a | 57.5 | 60 | 61 | 39 | 60 | 43 | 39 | 42 | 54 | 37 | 41 | 37 | 44 | 36 | 54 | 46 | 61.6 | 57.0 | 59.4 | 58.1 |
| 7b | 56.2 | 63 | 64 | 33 | 70 | 49 | 44 | 47 | 59 | 39 | 43 | 45 | 50 | 42 | 55 | 48 | 59.9 | 53.4 | 57.0 | 56.3 |
| 7c | 57.4 | 69 | 68 | 37 | 67 | 54 | 43 | 48 | 58 | 45 | 44 | 39 | 51 | 39 | 61 | 47 | 59.2 | 53.6 | 55.2 | 54.4 |
| 8a | 56.8 | 62 | 66 | 38 | 64 | 54 | 45 | 49 | 63 | 47 | 48 | 39 | 50 | 43 | 57 | 51 | 63.1 | 61.2 | 58.7 | 58.0 |
| 8b | 57.1 | 69 | 67 | 42 | 71 | 53 | 45 | 52 | 63 | 49 | 50 | 42 | 54 | 49 | 61 | 55 | 62.0 | 59.2 | 55.4 | 54.2 |
| 8c | 57.7 | 68 | 73 | 44 | 72 | 55 | 48 | 53 | 63 | 47 | 52 | 45 | 54 | 46 | 64 | 54 | 59.2 | 58.1 | 52.3 | 53.8 |
| 9a | 55.4 | 55 | 58 | 44 | 66 | 50 | 38 | 42 | 57 | 41 | 45 | 39 | 44 | 45 | 57 | 51 | 59.0 | 60.3 | 53.3 | 50.5 |
| 9b | 56.6 | 64 | 59 | 43 | 65 | 53 | 40 | 46 | 59 | 43 | 43 | 40 | 44 | 46 | 59 | 53 | 56.7 | 60.0 | 51.4 | 49.7 |
| 9c | 57.0 | 66 | 65 | 43 | 72 | 59 | 40 | 46 | 59 | 44 | 45 | 39 | 41 | 49 | 64 | 56 | 55.3 | 58.6 | 48.1 | 46.9 |
| 10a | 56.5 | 71 | 69 | 41 | 71 | 46 | 49 | 52 | 57 | 43 | 48 | 48 | 42 | 48 | 71 | 45 | 62.6 | 62.2 | 57.9 | 56.0 |
| 10b | 56.4 | 71 | 64 | 39 | 77 | 45 | 51 | 52 | 55 | 45 | 43 | 48 | 44 | 43 | 63 | 45 | 61.4 | 62.0 | 54.7 | 52.8 |
| 10c | 56.2 | 69 | 67 | 43 | 71 | 41 | 50 | 47 | 50 | 48 | 43 | 46 | 42 | 39 | 67 | 47 | 60.4 | 61.1 | 50.6 | 51.0 |
| 11a | 58.5 | 76 | 72 | 50 | 75 | 58 | 54 | 57 | 66 | 56 | 53 | 51 | 48 | 51 | 76 | 56 | 65.7 | 68.7 | 55.1 | 54.3 |
| 11b | 55.9 | 77 | 73 | 48 | 73 | 52 | 54 | 59 | 59 | 54 | 49 | 54 | 48 | 51 | 76 | 51 | 64.4 | 63.1 | 52.8 | 52.4 |
| 11c | 56.7 | 76 | 64 | 47 | 70 | 48 | 54 | 56 | 50 | 56 | 51 | 48 | 46 | 51 | 70 | 45 | 63.2 | 61.3 | 50.4 | 51.1 |
| 12a | 56.3 | 72 | 67 | 50 | 72 | 51 | 51 | 48 | 54 | 45 | 48 | 43 | 47 | 51 | 68 | 54 | 60.9 | 59.9 | 51.3 | 52.0 |
| 12b | 57.4 | 74 | 64 | 42 | 67 | 52 | 52 | 47 | 52 | 45 | 51 | 48 | 42 | 46 | 68 | 54 | 61.2 | 58.7 | 48.7 | 49.6 |
| 12c | 56.2 | 74 | 65 | 48 | 64 | 46 | 54 | 44 | 52 | 44 | 45 | 45 | 40 | 49 | 66 | 51 | 58.7 | 57.0 | 46.2 | 46.0 |
| 13a | 66.3 | 62 | 78 | 50 | 70 | 54 | 46 | 59 | 66 | 61 | 56 | 39 | 62 | 54 | 66 | 55 | 65.2 | 66.9 | 67.6 | 70.5 |
| 13b | 66.1 | 61 | 80 | 54 | 74 | 57 | 50 | 56 | 68 | 63 | 55 | 44 | 62 | 56 | 73 | 61 | 63.7 | 64.6 | 66.0 | 69.0 |
| 13c | 64.8 | 69 | 82 | 59 | 76 | 59 | 56 | 57 | 70 | 63 | 56 | 43 | 65 | 59 | 75 | 59 | 63.0 | 63.5 | 63.7 | 69.1 |
| 14a | 64.8 | 68 | 80 | 57 | 74 | 62 | 50 | 57 | 71 | 59 | 59 | 42 | 64 | 56 | 70 | 62 | 67.7 | 67.3 | 66.8 | 71.0 |
| 14b | 64.3 | 71 | 85 | 64 | 74 | 66 | 56 | 62 | 73 | 65 | 63 | 45 | 64 | 65 | 73 | 68 | 67.5 | 66.8 | 66.8 | 70.7 |
| 14c | 65.7 | 76 | 85 | 67 | 79 | 66 | 61 | 66 | 74 | 68 | 62 | 49 | 67 | 65 | 72 | 70 | 64.5 | 64.3 | 64.7 | 68.0 |
| 15a | 64.3 | 63 | 78 | 56 | 70 | 62 | 48 | 56 | 65 | 57 | 57 | 44 | 60 | 55 | 62 | 62 | 66.1 | 63.8 | 62.0 | 67.2 |
| 15b | 64.3 | 67 | 79 | 61 | 72 | 65 | 46 | 57 | 70 | 57 | 57 | 47 | 57 | 61 | 66 | 70 | 64.4 | 62.9 | 59.6 | 64.9 |
| 15c | 64.2 | 73 | 79 | 65 | 78 | 67 | 53 | 59 | 73 | 62 | 60 | 45 | 55 | 66 | 71 | 73 | 62.8 | 62.3 | 58.4 | 63.3 |
| 16a | 64.7 | 75 | 82 | 60 | 74 | 58 | 64 | 53 | 68 | 65 | 56 | 51 | 61 | 59 | 76 | 58 | 71.4 | 67.6 | 65.9 | 69.8 |
| 16b | 65.1 | 74 | 81 | 59 | 77 | 57 | 59 | 55 | 71 | 66 | 57 | 53 | 57 | 59 | 78 | 60 | 71.2 | 67.2 | 65.4 | 69.4 |
| 16c | 64.9 | 73 | 76 | 61 | 74 | 57 | 61 | 53 | 67 | 61 | 56 | 62 | 56 | 60 | 80 | 56 | 69.8 | 65.6 | 63.6 | 67.6 |
| 17a | 65.5 | 78 | 86 | 67 | 83 | 68 | 65 | 61 | 75 | 68 | 66 | 58 | 52 | 65 | 82 | 71 | 74.4 | 68.6 | 66.7 | 70.1 |
| 17b | 66.6 | 79 | 81 | 65 | 82 | 70 | 61 | 61 | 74 | 64 | 63 | 66 | 52 | 63 | 78 | 69 | 72.8 | 67.6 | 64.7 | 68.5 |
| 17c | 65.6 | 78 | 82 | 65 | 80 | 62 | 63 | 55 | 71 | 65 | 58 | 63 | 50 | 67 | 81 | 65 | 71.5 | 65.4 | 62.5 | 66.3 |
| 18a | 66.4 | 77 | 81 | 67 | 80 | 71 | 64 | 60 | 68 | 64 | 62 | 53 | 51 | 69 | 76 | 72 | 71.4 | 65.5 | 63.5 | 65.6 |
| 18b | 67.9 | 76 | 79 | 66 | 78 | 68 | 63 | 58 | 70 | 61 | 58 | 61 | 52 | 63 | 74 | 71 | 70.8 | 64.4 | 62.0 | 63.8 |
| 18c | 65.5 | 75 | 79 | 64 | 77 | 63 | 61 | 54 | 64 | 55 | 58 | 66 | 50 | 66 | 78 | 64 | 69.6 | 62.7 | 60.0 | 61.2 |
| 19a | 60.6 | 54 | 61 | 45 | 64 | 45 | 41 | 43 | 57 | 48 | 47 | 36 | 56 | 46 | 56 | 48 | 66.0 | 62.8 | 64.9 | 62.5 |
| 19b | 60.1 | 62 | 65 | 50 | 66 | 46 | 41 | 48 | 62 | 44 | 51 | 42 | 54 | 50 | 59 | 51 | 64.6 | 61.9 | 63.0 | 60.6 |
| 19c | 60.7 | 65 | 68 | 54 | 70 | 51 | 48 | 49 | 65 | 49 | 50 | 49 | 54 | 49 | 64 | 55 | 62.7 | 59.9 | 61.1 | 57.3 |
| 20a | 59.9 | 58 | 69 | 52 | 67 | 57 | 44 | 45 | 61 | 46 | 55 | 44 | 53 | 47 | 59 | 58 | 66.3 | 63.2 | 65.1 | 61.4 |
| 20b | 60.8 | 66 | 70 | 57 | 69 | 59 | 48 | 48 | 64 | 48 | 52 | 49 | 55 | 56 | 60 | 56 | 65.6 | 62.3 | 64.0 | 60.3 |
| 20c | 60.2 | 67 | 76 | 56 | 74 | 63 | 56 | 52 | 68 | 50 | 56 | 49 | 54 | 55 | 68 | 61 | 64.4 | 60.8 | 61.6 | 57.1 |
| 21a | 59.9 | 58 | 68 | 45 | 62 | 58 | 44 | 47 | 56 | 41 | 48 | 40 | 44 | 53 | 55 | 57 | 60.5 | 57.6 | 60.4 | 56.0 |
| 21b | 59.9 | 59 | 72 | 53 | 64 | 61 | 45 | 47 | 61 | 41 | 44 | 39 | 47 | 54 | 63 | 61 | 59.6 | 56.5 | 57.6 | 53.5 |
| 21c | 58.8 | 63 | 72 | 58 | 66 | 63 | 50 | 46 | 60 | 49 | 50 | 48 | 46 | 59 | 64 | 60 | 57.2 | 54.5 | 55.0 | 51.5 |
| 22a | 58.8 | 72 | 68 | 51 | 75 | 55 | 56 | 48 | 64 | 56 | 51 | 53 | 50 | 52 | 70 | 55 | 62.4 | 60.0 | 61.1 | 61.5 |
| 22b | 58.7 | 70 | 65 | 50 | 74 | 50 | 58 | 46 | 63 | 54 | 47 | 53 | 53 | 48 | 74 | 50 | 61.1 | 59.5 | 60.3 | 60.4 |
| 22c | 58.0 | 71 | 63 | 47 | 69 | 51 | 55 | 46 | 60 | 53 | 48 | 57 | 50 | 48 | 75 | 50 | 59.7 | 57.3 | 57.6 | 56.6 |
| 23a | 60.6 | 77 | 78 | 59 | 76 | 61 | 59 | 59 | 70 | 59 | 55 | 66 | 54 | 57 | 79 | 64 | 64.2 | 64.0 | 60.8 | 62.9 |
| 23b | 60.6 | 73 | 77 | 59 | 78 | 63 | 59 | 55 | 65 | 57 | 55 | 65 | 51 | 60 | 77 | 59 | 62.8 | 62.9 | 59.9 | 61.3 |
| 23c | 59.6 | 73 | 72 | 54 | 74 | 58 | 63 | 56 | 63 | 52 | 57 | 61 | 48 | 61 | 80 | 59 | 60.9 | 59.9 | 56.3 | 60.1 |
| 24a | 57.2 | 69 | 72 | 59 | 71 | 65 | 52 | 56 | 60 | 53 | 45 | 52 | 46 | 60 | 71 | 62 | 57.9 | 58.3 | 55.3 | 58.8 |
| 24b | 58.3 | 70 | 72 | 60 | 68 | 63 | 55 | 54 | 60 | 50 | 50 | 60 | 44 | 60 | 73 | 62 | 57.9 | 58.2 | 53.4 | 57.3 |
| 24c | 58.5 | 67 | 68 | 55 | 64 | 57 | 52 | 51 | 56 | 51 | 46 | 56 | 41 | 56 | 73 | 55 | 55.9 | 56.7 | 50.5 | 53.9 |

CWBC = Camera White Balance Calibration; PPWBC = Post-Processing White Balance Calibration
