# Supplementary material for: Color variations during digital imaging of facial prostheses subjected to unfiltered ambient light and image calibration techniques within dental clinics: An in vitro analysis
Source: PLoS One. 2022 Aug 29;17(8):e0273029. doi: 10.1371/journal.pone.0273029 (PMC9423681; doi:10.1371/journal.pone.0273029)
Supplement: S2 Table — (DOCX) [file pone.0273029.s002.docx]

# S2 Table. a* values from the pigmented silicone samples

| **Sample measurement** | **Spectro-photometer** | **Images without any white balance corrections  (Raw images)** | | | | | **CWBC** | | | | | **PPWBC using gray card** | | | | | **PPWBC using Macbeth color chart** | | | |
| --- | --- | --- | --- | --- | --- | --- | --- | --- | --- | --- | --- | --- | --- | --- | --- | --- | --- | --- | --- | --- |
|  |  | Photo box | Windowless  clinic 1 | Windowless clinic 2 | Windowed clinic 1 | Windowed clinic 2 | Photo box | Windowless clinic 1 | Windowless clinic 2 | Windowed clinic 1 | Windowed clinic 2 | Photo box | Windowless clinic 1 | Windowless clinic 2 | Windowed clinic 1 | Windowed clinic 2 | Windowless clinic 1 | Windowless clinic 2 | Windowed clinic 1 | Windowed clinic 2 |
| 1a | 8.6 | 20 | 10 | 9 | 13 | 11 | 20 | 9 | 9 | 10 | 11 | 14 | 13 | 12 | 13 | 13 | 12.2 | 7.3 | 8.4 | 7.9 |
| 1b | 8.3 | 19 | 7 | 9 | 11 | 11 | 21 | 9 | 9 | 11 | 11 | 15 | 12 | 11 | 12 | 13 | 10.8 | 10.0 | 6.2 | 8.1 |
| 1c | 8.4 | 19 | 9 | 9 | 10 | 9 | 22 | 8 | 9 | 9 | 9 | 16 | 11 | 12 | 13 | 13 | 11.4 | 7.8 | 7.0 | 6.8 |
| 2a | 8.2 | 20 | 8 | 10 | 10 | 10 | 21 | 9 | 9 | 11 | 9 | 16 | 13 | 13 | 14 | 13 | 12.1 | 7.6 | 8.0 | 8.3 |
| 2b | 8.4 | 19 | 9 | 10 | 10 | 10 | 22 | 8 | 8 | 10 | 10 | 17 | 12 | 12 | 12 | 13 | 12.4 | 6.3 | 6.8 | 7.9 |
| 2c | 8.2 | 15 | 9 | 10 | 9 | 9 | 22 | 8 | 10 | 10 | 8 | 15 | 14 | 13 | 11 | 10 | 12.1 | 8.5 | 8.7 | 6.4 |
| 3a | 8.9 | 22 | 10 | 10 | 10 | 10 | 23 | 10 | 10 | 9 | 11 | 17 | 13 | 13 | 16 | 12 | 12.4 | 9.2 | 8.6 | 9.0 |
| 3b | 8.7 | 20 | 10 | 10 | 11 | 10 | 23 | 9 | 11 | 10 | 10 | 16 | 14 | 14 | 13 | 14 | 10.8 | 7.8 | 8.0 | 6.8 |
| 3c | 8.7 | 19 | 8 | 9 | 8 | 9 | 20 | 10 | 10 | 10 | 8 | 15 | 12 | 13 | 15 | 11 | 12.3 | 7.6 | 8.1 | 7.9 |
| 4a | 8.8 | 17 | 8 | 9 | 10 | 11 | 22 | 10 | 10 | 10 | 10 | 17 | 13 | 13 | 13 | 13 | 8.2 | 7.8 | 8.4 | 8.2 |
| 4b | 8.8 | 14 | 8 | 9 | 11 | 13 | 21 | 9 | 10 | 11 | 10 | 18 | 11 | 14 | 13 | 12 | 6.8 | 6.9 | 8.7 | 8.3 |
| 4c | 8.6 | 15 | 8 | 8 | 9 | 8 | 21 | 7 | 7 | 7 | 11 | 17 | 11 | 11 | 10 | 12 | 7.5 | 6.5 | 6.4 | 6.9 |
| 5a | 8.2 | 15 | 7 | 8 | 8 | 7 | 21 | 8 | 7 | 9 | 8 | 14 | 11 | 11 | 9 | 10 | 6.7 | 6.1 | 6.0 | 5.7 |
| 5b | 8.2 | 15 | 7 | 9 | 8 | 10 | 21 | 8 | 8 | 8 | 8 | 16 | 12 | 11 | 11 | 11 | 7.4 | 7.3 | 8.1 | 7.1 |
| 5c | 8.4 | 15 | 7 | 8 | 10 | 8 | 22 | 8 | 8 | 10 | 9 | 14 | 12 | 13 | 10 | 11 | 7.1 | 7.6 | 7.5 | 8.7 |
| 6a | 8.5 | 16 | 8 | 9 | 10 | 9 | 22 | 8 | 9 | 9 | 8 | 16 | 12 | 13 | 12 | 11 | 7.2 | 7.5 | 7.8 | 6.3 |
| 6b | 8.5 | 16 | 9 | 9 | 8 | 11 | 22 | 10 | 9 | 10 | 11 | 17 | 13 | 12 | 12 | 14 | 7.2 | 8.0 | 8.2 | 8.0 |
| 6c | 8.6 | 16 | 9 | 9 | 11 | 10 | 23 | 9 | 10 | 10 | 10 | 16 | 13 | 13 | 12 | 14 | 7.9 | 8.1 | 8.0 | 8.9 |
| 7a | 3.6 | 25 | 8 | 7 | 12 | 9 | 21 | 7 | 8 | 9 | 9 | 14 | 11 | 10 | 14 | 13 | 6.3 | 6.1 | 7.0 | 6.8 |
| 7b | 3.7 | 23 | 8 | 7 | 10 | 9 | 21 | 8 | 7 | 9 | 8 | 15 | 10 | 10 | 14 | 12 | 6.5 | 6.1 | 6.8 | 7.1 |
| 7c | 3.6 | 23 | 7 | 6 | 12 | 8 | 22 | 7 | 7 | 11 | 9 | 15 | 10 | 10 | 14 | 13 | 6.7 | 6.7 | 6.9 | 6.7 |
| 8a | 3.7 | 24 | 8 | 8 | 11 | 9 | 21 | 7 | 8 | 10 | 9 | 15 | 10 | 10 | 14 | 13 | 6.0 | 6.6 | 6.2 | 6.4 |
| 8b | 3.7 | 21 | 7 | 7 | 10 | 9 | 21 | 7 | 7 | 9 | 8 | 15 | 10 | 10 | 15 | 13 | 6.3 | 6.7 | 6.8 | 6.8 |
| 8c | 3.8 | 23 | 7 | 8 | 10 | 10 | 23 | 7 | 8 | 10 | 10 | 15 | 10 | 11 | 14 | 14 | 6.7 | 6.9 | 6.7 | 6.3 |
| 9a | 3.5 | 23 | 7 | 6 | 10 | 7 | 18 | 5 | 6 | 9 | 8 | 12 | 9 | 9 | 12 | 12 | 5.6 | 5.3 | 3.3 | 5.2 |
| 9b | 3.7 | 23 | 8 | 7 | 10 | 8 | 20 | 6 | 7 | 9 | 8 | 14 | 11 | 10 | 13 | 12 | 6.0 | 5.8 | 5.9 | 5.8 |
| 9c | 3.8 | 24 | 8 | 7 | 11 | 10 | 22 | 7 | 7 | 11 | 9 | 15 | 10 | 11 | 14 | 13 | 6.1 | 6.2 | 5.9 | 6.4 |
| 10a | 3.3 | 19 | 4 | 5 | 8 | 8 | 21 | 6 | 6 | 8 | 8 | 13 | 8 | 8 | 12 | 11 | 4.3 | 4.8 | 4.7 | 4.3 |
| 10b | 3.1 | 20 | 6 | 6 | 5 | 6 | 21 | 6 | 6 | 8 | 7 | 14 | 9 | 10 | 11 | 10 | 4.9 | 5.4 | 4.6 | 5.0 |
| 10c | 3.3 | 21 | 5 | 6 | 8 | 6 | 21 | 5 | 5 | 7 | 7 | 14 | 8 | 9 | 11 | 9 | 4.4 | 4.9 | 4.8 | 4.5 |
| 11a | 3.5 | 19 | 7 | 8 | 9 | 8 | 22 | 7 | 8 | 10 | 10 | 16 | 11 | 11 | 14 | 13 | 6.8 | 4.4 | 6.6 | 6.8 |
| 11b | 3.5 | 18 | 6 | 6 | 8 | 7 | 22 | 7 | 5 | 8 | 7 | 15 | 9 | 10 | 11 | 11 | 5.0 | 5.3 | 5.2 | 5.3 |
| 11c | 3.3 | 18 | 7 | 5 | 9 | 6 | 22 | 6 | 6 | 5 | 6 | 15 | 9 | 9 | 11 | 10 | 4.0 | 4.4 | 4.9 | 4.6 |
| 12a | 3.4 | 21 | 7 | 8 | 9 | 9 | 21 | 7 | 7 | 9 | 9 | 13 | 8 | 11 | 11 | 13 | 5.6 | 5.2 | 5.6 | 4.8 |
| 12b | 3.4 | 19 | 7 | 6 | 9 | 8 | 20 | 6 | 7 | 10 | 6 | 14 | 9 | 10 | 10 | 11 | 4.4 | 5.2 | 5.1 | 5.1 |
| 12c | 3.3 | 19 | 6 | 5 | 10 | 7 | 22 | 5 | 6 | 7 | 6 | 14 | 8 | 9 | 12 | 10 | 4.6 | 4.8 | 5.0 | 6.0 |
| 13a | 8.9 | 23 | 9 | 11 | 11 | 12 | 22 | 10 | 10 | 12 | 12 | 14 | 14 | 14 | 15 | 16 | 9.4 | 9.0 | 9.7 | 9.5 |
| 13b | 8.6 | 24 | 9 | 11 | 12 | 13 | 22 | 10 | 10 | 11 | 11 | 15 | 14 | 13 | 14 | 15 | 9.1 | 9.5 | 10.3 | 10.0 |
| 13c | 8.7 | 22 | 7 | 9 | 11 | 10 | 23 | 10 | 10 | 11 | 11 | 15 | 13 | 13 | 13 | 14 | 7.8 | 8.2 | 8.6 | 8.7 |
| 14a | 8.1 | 21 | 8 | 8 | 10 | 10 | 21 | 8 | 8 | 10 | 11 | 14 | 11 | 11 | 14 | 14 | 7.9 | 6.6 | 7.3 | 7.5 |
| 14b | 8.2 | 22 | 7 | 10 | 10 | 10 | 22 | 10 | 9 | 10 | 11 | 15 | 13 | 12 | 13 | 13 | 8.0 | 8.5 | 8.6 | 8.2 |
| 14c | 8.0 | 19 | 7 | 10 | 10 | 12 | 24 | 10 | 9 | 11 | 11 | 17 | 13 | 14 | 14 | 15 | 9.5 | 9.0 | 8.5 | 9.4 |
| 15a | 8.6 | 23 | 9 | 10 | 12 | 12 | 22 | 9 | 12 | 12 | 11 | 15 | 13 | 14 | 16 | 15 | 8.9 | 9.0 | 8.9 | 9.1 |
| 15b | 9.0 | 23 | 9 | 11 | 12 | 12 | 22 | 11 | 9 | 12 | 11 | 16 | 13 | 14 | 14 | 14 | 9.0 | 8.7 | 8.6 | 8.9 |
| 15c | 8.9 | 22 | 9 | 11 | 11 | 11 | 24 | 10 | 10 | 12 | 12 | 17 | 14 | 13 | 14 | 15 | 9.4 | 8.6 | 9.1 | 9.4 |
| 16a | 8.5 | 20 | 8 | 9 | 11 | 11 | 22 | 9 | 9 | 10 | 11 | 17 | 14 | 13 | 13 | 14 | 8.4 | 8.1 | 9.0 | 8.4 |
| 16b | 9.0 | 21 | 8 | 10 | 11 | 11 | 24 | 10 | 10 | 11 | 11 | 18 | 14 | 14 | 13 | 16 | 9.2 | 9.1 | 9.2 | 9.5 |
| 16c | 8.4 | 21 | 10 | 10 | 11 | 11 | 24 | 11 | 10 | 11 | 12 | 18 | 14 | 14 | 12 | 15 | 9.5 | 9.2 | 9.2 | 9.9 |
| 17a | 8.7 | 18 | 7 | 11 | 10 | 11 | 22 | 10 | 10 | 12 | 11 | 17 | 14 | 14 | 12 | 14 | 8.7 | 9.3 | 9.5 | 9.4 |
| 17b | 8.9 | 18 | 9 | 11 | 9 | 11 | 24 | 10 | 11 | 12 | 11 | 18 | 14 | 16 | 14 | 15 | 9.5 | 9.1 | 9.7 | 9.5 |
| 17c | 9.1 | 20 | 9 | 10 | 11 | 13 | 24 | 10 | 10 | 12 | 11 | 18 | 13 | 14 | 12 | 15 | 9.5 | 9.6 | 10.1 | 9.9 |
| 18a | 9.0 | 20 | 9 | 11 | 10 | 12 | 23 | 10 | 10 | 11 | 11 | 18 | 14 | 14 | 14 | 15 | 9.7 | 9.0 | 9.2 | 9.1 |
| 18b | 8.3 | 20 | 9 | 11 | 10 | 11 | 24 | 10 | 10 | 11 | 12 | 18 | 14 | 14 | 14 | 13 | 9.3 | 9.3 | 9.2 | 9.4 |
| 18c | 8.1 | 20 | 9 | 10 | 11 | 13 | 24 | 10 | 10 | 11 | 12 | 18 | 13 | 14 | 13 | 16 | 9.4 | 9.3 | 9.4 | 9.4 |
| 19a | 3.1 | 21 | 7 | 5 | 9 | 8 | 18 | 5 | 5 | 8 | 7 | 12 | 9 | 9 | 13 | 11 | 4.4 | 3.8 | 4.7 | 4.8 |
| 19b | 3.1 | 21 | 6 | 5 | 10 | 8 | 20 | 5 | 6 | 8 | 7 | 14 | 9 | 10 | 13 | 12 | 4.6 | 4.4 | 5.1 | 4.9 |
| 19c | 3.1 | 21 | 6 | 6 | 9 | 9 | 21 | 6 | 6 | 9 | 7 | 15 | 9 | 10 | 13 | 12 | 5.2 | 5.0 | 6.9 | 7.4 |
| 20a | 3.0 | 21 | 6 | 5 | 9 | 7 | 19 | 5 | 5 | 8 | 6 | 13 | 9 | 8 | 13 | 12 | 4.0 | 3.7 | 4.6 | 4.7 |
| 20b | 3.2 | 22 | 6 | 6 | 9 | 8 | 21 | 6 | 5 | 8 | 7 | 15 | 9 | 10 | 13 | 12 | 4.6 | 4.9 | 5.2 | 5.1 |
| 20c | 2.2 | 21 | 5 | 6 | 9 | 8 | 23 | 6 | 6 | 9 | 7 | 16 | 10 | 10 | 13 | 12 | 4.8 | 4.9 | 5.7 | 5.2 |
| 21a | 5.9 | 24 | 7 | 6 | 10 | 9 | 21 | 6 | 6 | 8 | 7 | 13 | 10 | 10 | 14 | 13 | 5.5 | 4.9 | 5.8 | 5.6 |
| 21b | 6.0 | 24 | 6 | 8 | 10 | 9 | 22 | 6 | 7 | 9 | 8 | 15 | 10 | 12 | 14 | 13 | 5.6 | 5.0 | 5.7 | 5.3 |
| 21c | 5.9 | 23 | 7 | 7 | 9 | 9 | 23 | 6 | 6 | 10 | 8 | 16 | 10 | 11 | 14 | 13 | 5.7 | 5.4 | 5.8 | 5.0 |
| 22a | 4.4 | 20 | 6 | 6 | 9 | 8 | 23 | 5 | 6 | 8 | 8 | 17 | 10 | 11 | 12 | 13 | 4.5 | 5.0 | 5.4 | 5.4 |
| 22b | 4.3 | 21 | 7 | 6 | 8 | 8 | 23 | 6 | 6 | 9 | 7 | 17 | 10 | 10 | 13 | 12 | 5.4 | 5.3 | 5.5 | 5.8 |
| 22c | 5.0 | 21 | 7 | 7 | 10 | 8 | 24 | 6 | 6 | 9 | 8 | 17 | 10 | 10 | 12 | 12 | 5.6 | 5.1 | 6.2 | 6.0 |
| 23a | 2.1 | 18 | 5 | 6 | 8 | 8 | 22 | 7 | 5 | 9 | 8 | 14 | 10 | 10 | 9 | 12 | 3.9 | 4.0 | 4.6 | 5.0 |
| 23b | 2.2 | 20 | 6 | 6 | 8 | 8 | 23 | 6 | 6 | 8 | 8 | 16 | 10 | 10 | 11 | 13 | 4.5 | 5.0 | 5.3 | 6.0 |
| 23c | 2.0 | 20 | 5 | 5 | 7 | 9 | 22 | 6 | 6 | 9 | 7 | 16 | 9 | 8 | 10 | 12 | 4.8 | 4.9 | 5.7 | 5.3 |
| 24a | 4.7 | 21 | 7 | 6 | 8 | 9 | 22 | 5 | 7 | 8 | 8 | 16 | 9 | 11 | 12 | 14 | 4.6 | 4.8 | 5.1 | 5.3 |
| 24b | 5.0 | 21 | 6 | 7 | 9 | 9 | 23 | 6 | 7 | 9 | 8 | 17 | 9 | 11 | 12 | 13 | 5.1 | 5.1 | 5.5 | 6.0 |
| 24c | 4.9 | 22 | 7 | 6 | 9 | 9 | 23 | 5 | 7 | 9 | 8 | 16 | 9 | 11 | 11 | 14 | 5.4 | 4.8 | 5.3 | 5.5 |

CWBC = Camera White Balance Calibration; PPWBC = Post-Processing White Balance Calibration
