# Supplementary material for: Color variations during digital imaging of facial prostheses subjected to unfiltered ambient light and image calibration techniques within dental clinics: An in vitro analysis
Source: PLoS One. 2022 Aug 29;17(8):e0273029. doi: 10.1371/journal.pone.0273029 (PMC9423681; doi:10.1371/journal.pone.0273029)
Supplement: S3 Table — (DOCX) [file pone.0273029.s003.docx]

# S3 Table. b* values from the pigmented silicone samples

| **Sample measurement** | **Spectro-photometer** | **Images without any white balance corrections  (Raw images)** | | | | | **CWBC** | | | | | **PPWBC using gray card** | | | | | **PPWBC using Macbeth color chart** | | | |
| --- | --- | --- | --- | --- | --- | --- | --- | --- | --- | --- | --- | --- | --- | --- | --- | --- | --- | --- | --- | --- |
|  |  | Photo box | Windowless clinic 1 | Windowless clinic 2 | Windowed clinic 1 | Windowed clinic 2 | Photo box | Windowless clinic 1 | Windowless clinic 2 | Windowed clinic 1 | Windowed clinic 2 | Photo box | Windowless clinic 1 | Windowless clinic 2 | Windowed clinic 1 | Windowed clinic 2 | Windowless clinic 1 | Windowless clinic 2 | Windowed clinic 1 | Windowed clinic 2 |
| 1a | 46.2 | 41 | 21 | 20 | 26 | 23 | 29 | 19 | 21 | 21 | 22 | 18 | 23 | 23 | 25 | 24 | 19.9 | 18.2 | 20.9 | 21.4 |
| 1b | 45.0 | 42 | 19 | 21 | 24 | 24 | 32 | 20 | 21 | 22 | 22 | 18 | 23 | 22 | 22 | 25 | 19.2 | 20.3 | 19.9 | 20.7 |
| 1c | 46.1 | 43 | 21 | 20 | 23 | 22 | 33 | 20 | 22 | 21 | 21 | 21 | 23 | 23 | 25 | 25 | 19.4 | 18.7 | 22.5 | 21.0 |
| 2a | 46.1 | 42 | 22 | 19 | 24 | 24 | 31 | 20 | 23 | 22 | 22 | 21 | 25 | 24 | 25 | 26 | 20.7 | 19.2 | 21.7 | 22.4 |
| 2b | 45.9 | 44 | 23 | 21 | 25 | 24 | 32 | 20 | 22 | 22 | 23 | 22 | 25 | 24 | 24 | 28 | 20.4 | 17.8 | 20.8 | 21.3 |
| 2c | 45.3 | 38 | 21 | 22 | 24 | 23 | 34 | 19 | 23 | 23 | 23 | 21 | 25 | 26 | 23 | 26 | 20.7 | 19.6 | 21.6 | 20.2 |
| 3a | 45.7 | 42 | 23 | 20 | 24 | 23 | 32 | 18 | 22 | 22 | 22 | 21 | 23 | 25 | 27 | 25 | 19.7 | 19.8 | 21.6 | 22.1 |
| 3b | 45.6 | 43 | 22 | 21 | 27 | 23 | 31 | 19 | 23 | 23 | 22 | 21 | 25 | 26 | 24 | 28 | 19.3 | 18.8 | 21.5 | 20.7 |
| 3c | 46.2 | 44 | 22 | 20 | 21 | 22 | 30 | 20 | 22 | 21 | 22 | 20 | 24 | 24 | 25 | 23 | 20.0 | 19.4 | 21.9 | 21.8 |
| 4a | 45.8 | 42 | 20 | 21 | 24 | 24 | 35 | 21 | 23 | 22 | 22 | 23 | 23 | 25 | 25 | 25 | 19.5 | 19.0 | 21.9 | 20.9 |
| 4b | 45.9 | 38 | 21 | 20 | 31 | 26 | 34 | 21 | 22 | 24 | 21 | 23 | 21 | 24 | 31 | 25 | 16.9 | 16.3 | 22.0 | 21.7 |
| 4c | 45.7 | 41 | 20 | 20 | 23 | 21 | 34 | 19 | 21 | 22 | 20 | 22 | 21 | 23 | 22 | 24 | 18.1 | 17.9 | 19.9 | 20.4 |
| 5a | 45.8 | 42 | 20 | 20 | 23 | 23 | 36 | 21 | 21 | 23 | 23 | 23 | 24 | 25 | 20 | 24 | 17.7 | 18.4 | 21.0 | 20.2 |
| 5b | 44.5 | 40 | 20 | 21 | 24 | 26 | 35 | 21 | 22 | 22 | 23 | 24 | 23 | 24 | 24 | 26 | 18.7 | 19.1 | 21.6 | 21.0 |
| 5c | 45.8 | 41 | 20 | 20 | 26 | 23 | 35 | 20 | 22 | 25 | 22 | 23 | 23 | 25 | 22 | 24 | 19.1 | 18.6 | 21.1 | 23.2 |
| 6a | 45.7 | 41 | 21 | 22 | 25 | 23 | 33 | 20 | 22 | 23 | 22 | 23 | 23 | 26 | 24 | 26 | 19.0 | 18.9 | 22.1 | 21.0 |
| 6b | 45.9 | 41 | 22 | 22 | 23 | 24 | 33 | 20 | 22 | 24 | 22 | 24 | 23 | 26 | 25 | 27 | 18.8 | 19.4 | 22.1 | 21.6 |
| 6c | 45.7 | 42 | 22 | 21 | 27 | 23 | 33 | 19 | 21 | 23 | 21 | 23 | 21 | 25 | 23 | 26 | 18.6 | 19.1 | 21.7 | 21.8 |
| 7a | 34.3 | 39 | 21 | 16 | 25 | 19 | 29 | 18 | 21 | 19 | 19 | 19 | 22 | 18 | 23 | 22 | 20.3 | 19.2 | 21.4 | 21.1 |
| 7b | 33.9 | 40 | 21 | 17 | 26 | 21 | 30 | 18 | 21 | 19 | 20 | 19 | 22 | 19 | 25 | 23 | 19.5 | 19.6 | 21.1 | 20.7 |
| 7c | 34.6 | 43 | 22 | 17 | 29 | 21 | 31 | 19 | 21 | 20 | 20 | 20 | 22 | 18 | 26 | 22 | 19.5 | 19.0 | 20.8 | 20.4 |
| 8a | 35.1 | 40 | 23 | 17 | 28 | 23 | 30 | 19 | 22 | 20 | 21 | 19 | 22 | 20 | 25 | 24 | 20.1 | 20.3 | 21.7 | 20.9 |
| 8b | 35.1 | 42 | 23 | 18 | 28 | 23 | 30 | 20 | 22 | 22 | 21 | 21 | 23 | 20 | 26 | 24 | 19.8 | 19.9 | 21.9 | 21.0 |
| 8c | 34.8 | 44 | 23 | 20 | 28 | 24 | 33 | 21 | 24 | 22 | 25 | 20 | 23 | 22 | 27 | 26 | 20.6 | 19.8 | 21.6 | 20.2 |
| 9a | 34.5 | 39 | 23 | 18 | 26 | 22 | 28 | 17 | 21 | 19 | 21 | 18 | 20 | 20 | 24 | 24 | 19.5 | 19.3 | 20.2 | 18.9 |
| 9b | 34.5 | 42 | 24 | 18 | 27 | 22 | 28 | 17 | 21 | 20 | 20 | 19 | 21 | 20 | 26 | 24 | 18.6 | 19.1 | 19.1 | 19.2 |
| 9c | 34.8 | 43 | 21 | 18 | 27 | 22 | 31 | 17 | 21 | 21 | 21 | 20 | 20 | 21 | 26 | 25 | 18.9 | 18.9 | 19.0 | 18.7 |
| 10a | 34.8 | 42 | 21 | 18 | 27 | 22 | 31 | 21 | 22 | 20 | 21 | 21 | 20 | 21 | 28 | 23 | 20.3 | 21.0 | 20.7 | 21.1 |
| 10b | 34.1 | 43 | 24 | 17 | 27 | 20 | 33 | 20 | 21 | 21 | 20 | 22 | 21 | 20 | 29 | 21 | 20.4 | 20.5 | 21.1 | 21.0 |
| 10c | 34.8 | 42 | 23 | 17 | 25 | 20 | 31 | 19 | 19 | 20 | 19 | 21 | 20 | 19 | 28 | 20 | 19.6 | 19.8 | 20.6 | 20.1 |
| 11a | 33.7 | 44 | 23 | 20 | 27 | 24 | 34 | 22 | 23 | 23 | 24 | 23 | 23 | 23 | 29 | 25 | 21.4 | 20.7 | 21.5 | 21.8 |
| 11b | 35.0 | 43 | 25 | 20 | 27 | 22 | 35 | 22 | 21 | 22 | 22 | 23 | 22 | 22 | 27 | 24 | 21.5 | 21.2 | 20.7 | 20.6 |
| 11c | 34.0 | 43 | 23 | 18 | 27 | 20 | 34 | 21 | 20 | 20 | 21 | 21 | 20 | 22 | 29 | 22 | 20.0 | 20.9 | 19.5 | 19.7 |
| 12a | 34.3 | 43 | 22 | 20 | 26 | 23 | 32 | 19 | 21 | 20 | 21 | 20 | 21 | 23 | 27 | 25 | 20.9 | 20.1 | 19.9 | 20.4 |
| 12b | 34.6 | 43 | 23 | 19 | 26 | 22 | 32 | 18 | 21 | 21 | 20 | 22 | 20 | 22 | 27 | 24 | 20.5 | 20.0 | 19.1 | 20.1 |
| 12c | 35.2 | 43 | 22 | 19 | 26 | 21 | 33 | 18 | 20 | 21 | 20 | 20 | 19 | 21 | 28 | 23 | 20.1 | 19.6 | 18.6 | 19.4 |
| 13a | 43.6 | 38 | 21 | 19 | 23 | 23 | 30 | 20 | 23 | 23 | 22 | 18 | 24 | 22 | 25 | 24 | 18.8 | 19.3 | 21.7 | 22.2 |
| 13b | 43.3 | 41 | 22 | 20 | 26 | 23 | 31 | 19 | 23 | 24 | 22 | 20 | 24 | 23 | 27 | 25 | 19.8 | 20.3 | 21.7 | 22.7 |
| 13c | 43.6 | 41 | 19 | 20 | 24 | 22 | 33 | 20 | 22 | 25 | 22 | 21 | 24 | 23 | 26 | 24 | 18.6 | 19.2 | 22.7 | 20.7 |
| 14a | 44.8 | 42 | 22 | 21 | 27 | 26 | 32 | 22 | 24 | 24 | 24 | 19 | 26 | 25 | 27 | 27 | 20.5 | 20.5 | 23.1 | 22.8 |
| 14b | 43.7 | 43 | 19 | 21 | 27 | 25 | 34 | 21 | 22 | 25 | 24 | 20 | 26 | 26 | 26 | 26 | 19.9 | 19.3 | 21.7 | 22.5 |
| 14c | 42.1 | 43 | 19 | 23 | 26 | 26 | 37 | 22 | 23 | 25 | 24 | 22 | 26 | 26 | 27 | 28 | 20.0 | 19.9 | 21.6 | 22.6 |
| 15a | 43.1 | 41 | 21 | 20 | 27 | 24 | 31 | 18 | 23 | 23 | 22 | 19 | 22 | 25 | 26 | 26 | 19.9 | 19.7 | 21.6 | 22.2 |
| 15b | 43.7 | 42 | 22 | 22 | 27 | 25 | 32 | 20 | 21 | 22 | 22 | 21 | 24 | 24 | 26 | 27 | 20.4 | 19.5 | 20.8 | 22.0 |
| 15c | 43.5 | 44 | 22 | 23 | 27 | 26 | 34 | 21 | 23 | 25 | 22 | 21 | 23 | 26 | 26 | 26 | 19.5 | 19.5 | 21.0 | 22.6 |
| 16a | 41.9 | 40 | 18 | 19 | 24 | 21 | 36 | 18 | 20 | 22 | 20 | 21 | 24 | 22 | 23 | 23 | 17.6 | 17.3 | 19.8 | 20.3 |
| 16b | 43.1 | 42 | 20 | 21 | 26 | 23 | 37 | 20 | 22 | 25 | 22 | 24 | 24 | 24 | 25 | 25 | 19.8 | 19.8 | 21.9 | 22.3 |
| 16c | 41.7 | 42 | 21 | 20 | 25 | 23 | 37 | 18 | 21 | 24 | 22 | 25 | 23 | 23 | 25 | 25 | 19.2 | 19.3 | 21.2 | 21.9 |
| 17a | 42.4 | 42 | 18 | 22 | 25 | 25 | 38 | 21 | 22 | 24 | 24 | 24 | 24 | 25 | 25 | 25 | 19.5 | 19.5 | 21.8 | 21.7 |
| 17b | 42.8 | 42 | 21 | 22 | 24 | 24 | 38 | 21 | 24 | 26 | 24 | 26 | 23 | 26 | 28 | 27 | 20.5 | 19.8 | 22.0 | 22.7 |
| 17c | 43.3 | 42 | 20 | 22 | 26 | 24 | 38 | 19 | 23 | 24 | 23 | 27 | 22 | 26 | 25 | 26 | 20.4 | 19.9 | 22.2 | 22.7 |
| 18a | 43.8 | 42 | 22 | 24 | 26 | 27 | 38 | 21 | 23 | 24 | 24 | 24 | 23 | 28 | 27 | 28 | 19.9 | 20.7 | 22.1 | 22.9 |
| 18b | 42.3 | 42 | 21 | 22 | 26 | 25 | 38 | 20 | 23 | 24 | 24 | 25 | 23 | 26 | 28 | 26 | 19.9 | 19.9 | 22.9 | 22.7 |
| 18c | 41.4 | 43 | 23 | 22 | 27 | 25 | 38 | 20 | 23 | 23 | 22 | 26 | 22 | 25 | 26 | 27 | 20.4 | 19.9 | 22.4 | 22.1 |
| 19a | 38.7 | 38 | 25 | 21 | 30 | 23 | 31 | 19 | 24 | 23 | 23 | 22 | 26 | 23 | 27 | 26 | 23.6 | 22.9 | 25.7 | 25.3 |
| 19b | 35.8 | 41 | 26 | 22 | 32 | 25 | 33 | 22 | 26 | 24 | 24 | 24 | 27 | 25 | 30 | 26 | 24.5 | 23.8 | 26.3 | 25.1 |
| 19c | 38.5 | 43 | 26 | 22 | 31 | 26 | 34 | 22 | 27 | 25 | 25 | 25 | 27 | 25 | 31 | 27 | 24.6 | 23.8 | 25.7 | 24.3 |
| 20a | 39.1 | 40 | 27 | 23 | 31 | 26 | 33 | 22 | 25 | 23 | 25 | 24 | 26 | 25 | 29 | 28 | 24.2 | 23.4 | 26.0 | 25.7 |
| 20b | 39.1 | 43 | 27 | 24 | 31 | 28 | 35 | 22 | 27 | 24 | 26 | 25 | 27 | 26 | 30 | 29 | 24.6 | 23.5 | 26.5 | 25.4 |
| 20c | 32.1 | 45 | 27 | 24 | 31 | 28 | 37 | 23 | 27 | 25 | 26 | 26 | 28 | 27 | 31 | 30 | 23.9 | 22.8 | 26.3 | 24.3 |
| 21a | 47.4 | 42 | 29 | 24 | 33 | 28 | 32 | 24 | 26 | 22 | 24 | 23 | 24 | 28 | 29 | 29 | 24.9 | 24.1 | 26.5 | 25.6 |
| 21b | 47.5 | 44 | 29 | 26 | 32 | 28 | 35 | 23 | 27 | 24 | 24 | 25 | 26 | 27 | 31 | 31 | 24.9 | 24.2 | 26.3 | 24.8 |
| 21c | 47.2 | 45 | 29 | 26 | 32 | 30 | 36 | 24 | 27 | 25 | 25 | 27 | 25 | 30 | 32 | 32 | 24.7 | 23.9 | 25.7 | 24.2 |
| 22a | 47.4 | 48 | 30 | 25 | 33 | 26 | 38 | 23 | 29 | 28 | 26 | 29 | 27 | 28 | 33 | 29 | 24.9 | 24.1 | 27.1 | 26.8 |
| 22b | 46.7 | 47 | 29 | 25 | 33 | 26 | 39 | 24 | 29 | 28 | 25 | 30 | 28 | 27 | 35 | 28 | 25.5 | 25.4 | 27.6 | 27.3 |
| 22c | 48.2 | 47 | 29 | 25 | 33 | 27 | 40 | 24 | 28 | 27 | 25 | 30 | 28 | 27 | 35 | 28 | 25.3 | 24.9 | 26.6 | 27.0 |
| 23a | 40.0 | 48 | 27 | 25 | 32 | 28 | 40 | 26 | 27 | 28 | 27 | 27 | 28 | 27 | 29 | 30 | 23.6 | 23.4 | 25.5 | 26.1 |
| 23b | 39.7 | 49 | 27 | 24 | 31 | 28 | 41 | 27 | 27 | 27 | 26 | 31 | 28 | 28 | 32 | 29 | 24.1 | 23.4 | 24.8 | 25.4 |
| 23c | 39.3 | 48 | 27 | 25 | 32 | 28 | 41 | 26 | 27 | 27 | 25 | 30 | 26 | 27 | 31 | 29 | 23.9 | 24.0 | 25.1 | 25.3 |
| 24a | 47.1 | 47 | 30 | 28 | 32 | 31 | 38 | 23 | 28 | 26 | 25 | 28 | 24 | 30 | 34 | 32 | 24.0 | 24.5 | 24.8 | 26.8 |
| 24b | 48.0 | 48 | 30 | 28 | 33 | 30 | 39 | 23 | 28 | 26 | 25 | 30 | 23 | 30 | 34 | 32 | 24.4 | 24.6 | 25.4 | 27.0 |
| 24c | 48.0 | 47 | 29 | 26 | 33 | 29 | 38 | 22 | 27 | 26 | 25 | 29 | 23 | 30 | 33 | 30 | 24.5 | 24.3 | 24.9 | 25.6 |

CWBC = Camera White Balance Calibration; PPWBC = Post-Processing White Balance Calibration
