# Supplementary material for: Color variations during digital imaging of facial prostheses subjected to unfiltered ambient light and image calibration techniques within dental clinics: An in vitro analysis
Source: PLoS One. 2022 Aug 29;17(8):e0273029. doi: 10.1371/journal.pone.0273029 (PMC9423681; doi:10.1371/journal.pone.0273029)
Supplement: S4 Table — (DOCX) [file pone.0273029.s004.docx]

# S4 Table. Dunn’s test results at different environmental lighting conditions

| **Environmental conditions** | **Images without any white balance corrections  (Raw images)** | | | **CWBC** | | | **PPWBC using  gray card** | | | **PPWBC using  Macbeth color chart** | | |
| --- | --- | --- | --- | --- | --- | --- | --- | --- | --- | --- | --- | --- |
|  | ***L*** | ***a*** | ***b*** | ***L*** | ***a*** | ***b*** | ***L*** | ***a*** | ***b*** | ***L*** | ***a*** | ***b*** |
| Spectrophotometer  *vs.* Windowless clinic 2 | * | * | * | NS | * | * | * | * | * | NS | ** | * |
| Spectrophotometer  *vs.*  photo box | * | * | NS | * | * | * | * | * | * | * | ** | * |
| Spectrophotometer  *vs.*  Windowed clinic 1 | * | * | * | * | * | * | * | * | * | NS | ** | * |
| Spectrophotometer  *vs.*  Windowless clinic 1 | * | NS | * | * | * | * | * | * | * | * | ** | * |
| Spectrophotometer  *vs.*  Windowed clinic 2 | NS | * | * | * | * | * | NS | * | * | NS | ** | * |
| Windowless clinic 2  *vs.*  Windowed clinic 2 | * | * | * | * | * | NS | * | * | * | NS | ** | * |
| Windowless clinic 2  *vs.*  Windowed clinic 1 | * | * | * | * | * | NS | * | * | * | NS | ** | * |
| Windowless clinic 2  *vs.*  Windowless clinic 1 | * | NS | * | * | NS | * | NS | NS | NS | NS | ** | NS |
| Windowed clinic 2  *vs.*  Windowed clinic 1 | * | NS | * | NS | NS | NS | * | NS | NS | NS | ** | NS |
| Windowed clinic 2  *vs.*  Windowless clinic 1 | * | * | NS | NS | * | * | * | * | * | * | ** | * |
| Windowed clinic 1  *vs.*  Windowless clinic 1 | NS | * | * | NS | * | * | * | * | * | * | ** | * |
| Windowless clinic 2  *vs.*  photo box | * | * | * | * | * | * | * | * | * | * | ** | * |
| Windowed clinic 2  *vs.*  photo box | * | * | * | NS | * | * | * | * | * | * | ** | * |
| Photo box  *vs.*  Windowed clinic 1 | NS | * | * | * | * | * | * | * | * | * | ** | * |
| Photo box  *vs.*  Windowless clinic 1 | NS | * | * | NS | * | * | NS | * | NS | * | ** | * |

*Significant differences (P<.05); CWBC = Camera White Balance Calibration; PPWBC = Post-Processing White Balance Calibration

** Null hypothesis for a* values are accepted
