# Supplementary material for: Color variations during digital imaging of facial prostheses subjected to unfiltered ambient light and image calibration techniques within dental clinics: An in vitro analysis
Source: PLoS One. 2022 Aug 29;17(8):e0273029. doi: 10.1371/journal.pone.0273029 (PMC9423681; doi:10.1371/journal.pone.0273029)
Supplement: S5 Table — (DOCX) [file pone.0273029.s005.docx]

# S5 Table. Color differences (∆E) produced between different methods of color calibration in relevant clinical lighting scenarios

| **Type of clinic** | **Method 1** | **∆E*** | **Method 2** |
| --- | --- | --- | --- |
| Windowless clinic | CWBC | 6.46 | PPWBC using 18% grey card |
|  | PPWBC using 18% grey card | 10.87 | PPWBC using Macbeth color chart |
|  | PPWBC using Macbeth color chart | 6.69 | CWBC |
| Windowed clinic | CWBC | 12.02 | PPWBC using 18% grey card |
|  | PPWBC using 18% grey card | 9.59 | PPWBC using Macbeth color chart |
|  | PPWBC using Macbeth color chart | 7.29 | CWBC |

* ∆E represents the color differences produced when comparing Method 1 VS Method 2;
CWBC = Camera White Balance Calibration; PPWBC = Post-Processing White Balance Calibration
